# Sustainable Polymer Additive Manufacturing Across Scales: A Critical Review of Pallet-Scale Structural Opportunities and Membrane Feed Spacers

**DOI:** 10.3390/polym18182208

**Published:** 2026-09-10

**Authors:** Anil Bairapudi, M. Venkata Kishore, B. Veera Siva Reddy, C. Chandrasekhara Sastry, Robert Cep

**Affiliations:** 1 Department of Mechanical Engineering, Indian Institute of Information Technology Design and Manufacturing Kurnool (IIITDM Kurnool), Kurnool 518008, Andhra Pradesh, India; 2 Central Institute of Tool Design (CITD), IDA, Balanagar, Hyderabad 500037, Telangana, India; 3 Department of Machining, Assembly and Engineering Metrology, Faculty of Mechanical Engineering, VSB-Technical University of Ostrava, 70800 Ostrava, Czech Republic

**Keywords:** additive manufacturing, sustainability, FDM, SLA, DLP, pallets, feed spacers, recycling, lifecycle, circularity

## Abstract

Additive manufacturing (AM) can support more sustainable polymer production, but the benefit is conditional on process energy, material chemistry, build yield, post-processing, service life, repair, and end-of-life recovery. This critical integrative review compares three polymer AM routes: stereolithography (SLA), digital light processing (DLP), and fused deposition modelling (FDM) through two deliberately contrasting application scales: pallet-scale load-bearing structures and membrane feed spacers. The review distinguishes direct application evidence from design opportunities inferred from adjacent AM literature. For pallet-scale structures, the literature currently supports large-format thermoplastic extrusion, zoned cellular architectures, modular repair, and controlled recycled feedstock as plausible translation routes, but direct peer-reviewed evidence for fully additively manufactured transportation pallets remains very limited. For membrane feed spacers, direct studies provide stronger quantitative evidence: published 3D-printed designs have reported approximately threefold pressure-drop reduction with doubled specific water flux, pressure-drop gradients as low as 0.091 bar m^−1^ under reported test conditions, and a 16% increase in permeate flux with a thinner fouling layer for a honeycomb geometry. Process-energy evidence also shows that results depend strongly on the functional unit and machine state; reported desktop values span 24.8–85.7 kJ cm^−3^ for FFF and 10.8–21.5 kJ cm^−3^ for SLA, while post-processing and machine utilization can materially change the lifecycle result. Recycled polymers likewise involve a performance–circularity trade-off: some post-consumer PLA studies report strength losses of about one-third or more, whereas controlled blends and recycling strategies can retain a much larger fraction of virgin-material performance. The synthesis therefore treats geometry, process parameters, material state, operational performance, lifecycle impact, and cost as one coupled design problem rather than assuming that AM, recycled content, or bio-based chemistry is inherently sustainable.

## 1. Introduction

Sustainable manufacturing has shifted from a peripheral environmental consideration to a design and production requirement that increasingly influences materials selection, process planning, supply-chain configuration, product architecture, and end-of-life strategy. Conventional subtractive and forming routes remain indispensable for high-volume manufacturing, but they can involve substantial material removal, tooling, coolant use, process scrap, and centralized supply chains. AM changes this relationship by fabricating geometry directly from a digital model and by placing material only where the design requires it. The sustainability opportunity therefore arises not simply from reduced chip formation, but from a wider combination of near-net-shape production, part consolidation, geometry-enabled lightweighting, distributed manufacturing, reduced inventory, repair, and the potential use of recycled or renewable feedstocks [1,2,3,4,5].

At the same time, a critical review must avoid the assumption that AM is automatically greener than conventional manufacturing. Energy demand per unit mass can be high, especially when machines operate below full build utilization or when long build times, thermal conditioning, support removal, solvent washing, post-curing, or thermal post-processing are required. Polymer and photopolymer feedstocks also introduce different environmental burdens: thermoplastics may be recyclable but can undergo thermal degradation during repeated processing, whereas vat-photopolymerization resins can deliver exceptional detail but often involve reactive monomers, photoinitiators, solvent cleaning, and more complex end-of-life handling. Accordingly, the environmental value of AM is application-dependent and should be judged over the product lifecycle rather than at the printing stage alone [1,5,6,7,8,9,10].

This application dependence is especially visible when comparing pallet-scale logistics structures with membrane feed spacers. Pallets are large load-bearing products whose performance is governed by stiffness, impact resistance, repeated handling, creep, dimensional stability, repairability, and compatibility with warehouse systems. The AM sustainability opportunity at this scale lies in structural lightweighting, local reinforcement, modular replacement, recycled thermoplastics, and large-format extrusion, but these opportunities should not be presented as demonstrated whole-pallet performance where direct evidence is absent. Feed spacers, by contrast, are small but functionally critical components within spiral-wound membrane modules. Their performance is dictated by channel geometry, local shear, mixing, mass transfer, pressure drop, fouling, cleaning, chemical stability, and manufacturable feature size. Here, sustainability is linked not only to material use but also to pumping energy, cleaning demand, water productivity, and membrane life [11,12,13,14,15,16,17].

Considering these two components together is scientifically useful because they expose the central tension in sustainable AM: the same manufacturing platform must be evaluated against very different service-dominant performance metrics. In a pallet, structural efficiency and production scale may dominate. In a feed spacer, geometric fidelity, surface chemistry, and fluid-mechanical effects may dominate. A process that is attractive for one application can therefore be inappropriate for the other. FDM is accessible, scalable, repairable, and compatible with thermoplastic recycling, but it has pronounced interlayer anisotropy and finite feature resolution. SLA and DLP produce smoother and finer geometries, but resin handling, post-curing, material brittleness, and recyclability require careful consideration [18,19,20,21,22,23,24,25].

Recent material studies reinforce that sustainable polymer AM must be judged by retained performance, processing burden, service conditions, and end-of-life pathway rather than by recycled or bio-based content alone. Recycled feedstocks can undergo property changes during reprocessing, while renewable photopolymer systems can still require energy-intensive curing and remain difficult to recycle after cross-linking [6,7,8,9,10]. These trade-offs motivate the application-specific framework developed here.

The same conditional logic is visible across sectors. Aerospace and automotive applications can justify AM through lightweighting, low-volume customization, or consolidated tooling; healthcare can justify it through patient-specific geometry; electronics and packaging can benefit from integrated housings and embedded functions; and water treatment can exploit controlled channels and surfaces [26,27,28,29,30,31,32,33]. These examples are useful only when the service benefit is measured against machine energy, material production, post-processing, rejection, maintenance, and recovery. The present review therefore uses pallets and feed spacers as two boundary cases rather than as the only sectors in which sustainable polymer AM is relevant.

The literature represented in this review spans sustainability assessment, SLA/DLP chemistry, ceramic and nanocomposite printing, FDM parameter optimization, recycled filament, membrane fabrication, water-treatment structures, circular economy, and Industry 4.0. Rather than treating these topics as separate technology summaries, the present review synthesizes them around four questions: (i) which AM process characteristics are most relevant to sustainable pallet and spacer design; (ii) how do material, geometry, and process parameters interact with application performance; (iii) where can lifecycle benefits outweigh manufacturing burdens; and (iv) what evidence is still required for credible industrial translation? This structure converts a technology-centred literature survey into an application-centred critical review [3,10,34,35].

**Figure 1:**
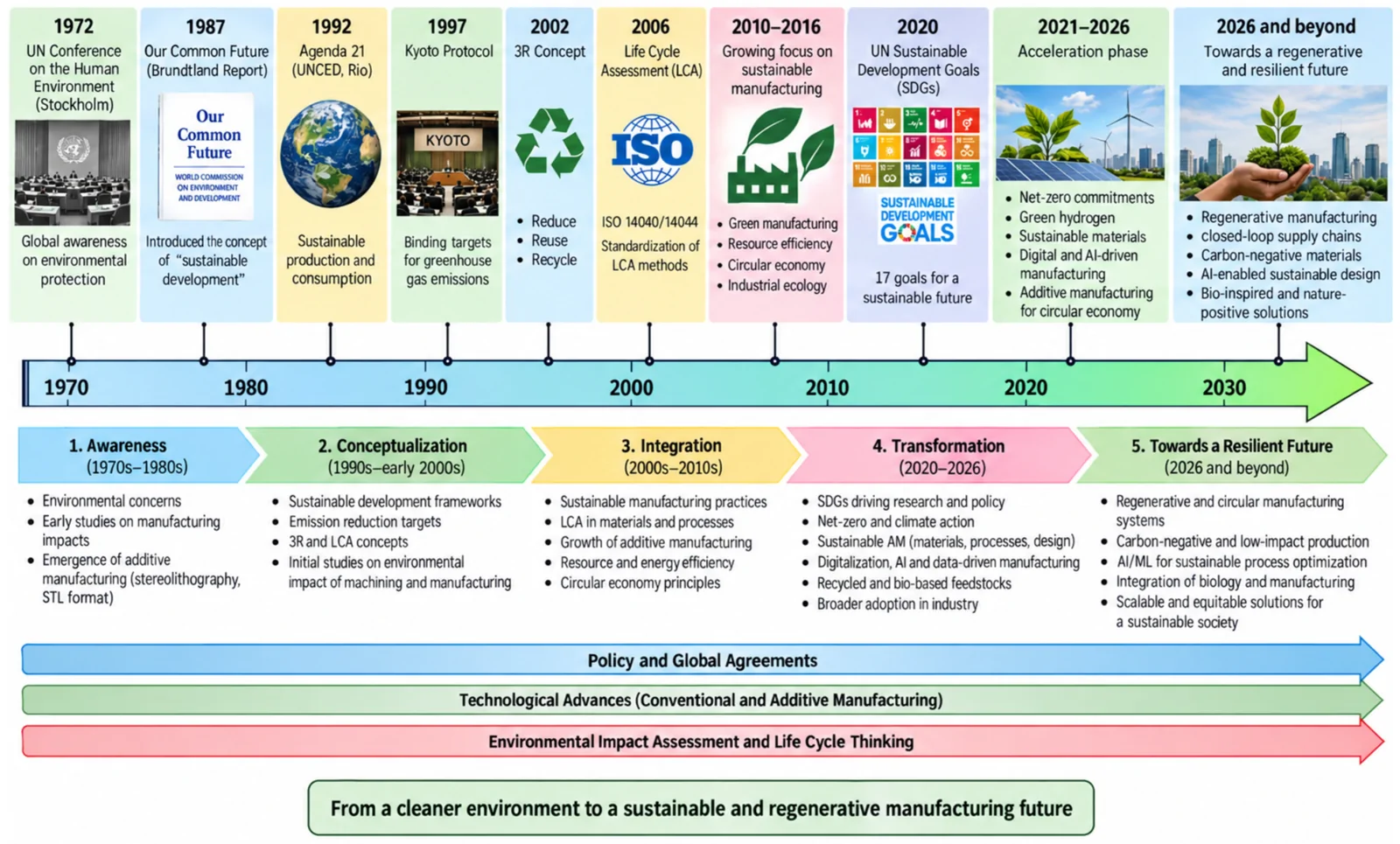
Timeline of sustainable manufacturing milestones [1].

Figure 1 places AM within the broader evolution of sustainable manufacturing. The key implication of this trajectory is that sustainability has progressively expanded from pollution control and waste minimization toward lifecycle thinking, circularity, digital integration, and design-stage intervention. AM fits this evolution particularly well because it connects digital geometry with physical resource use. However, that connection yields meaningful environmental improvement only when the design exploits AM-specific capabilities rather than reproducing a conventional part using a different manufacturing process [1,2,34].

The novelty of this review lies in comparing two intentionally asymmetric evidence domains under one process–material–function–lifecycle framework. Pallet-scale structures represent a macro-scale, structure-dominated translation problem in which mass, stiffness, impact, creep, repair, production rate, and material recovery govern viability; the available evidence is predominantly enabling rather than direct. Membrane feed spacers represent a smaller, transport-dominated application in which primary AM studies directly connect printed geometry with pressure drop, flux, fouling, and biofilm behaviour. Treating these evidence levels separately prevents design opportunities from being presented as demonstrated component performance and allows process selection to be judged against service function rather than printing capability alone.

## 2. Review Scope, Literature Selection, and Analytical Framework

This article is a critical integrative narrative review, not a formal systematic review [36,37,38]. The literature-identification process was revised to make that distinction explicit. Targeted searches were performed in Scopus and Web of Science and were supplemented by Google Scholar for forward and backward citation chaining. The primary search window was 2000 to August 2026, with earlier seminal studies retained when they establish foundational pallet mechanics, spacer hydrodynamics, cellular-structure design, or AM process behaviour. English-language peer-reviewed journal articles and reviews were prioritized; selected books and conference papers were retained only for foundational concepts not adequately represented elsewhere.

Search terms were organized in linked concept blocks rather than one universal string. The AM block included “additive manufacturing”, “3D printing”, “fused deposition modelling”, “fused filament fabrication”, “stereolithography”, “digital light processing”, “large-format additive manufacturing”, “lattice”, and “TPMS”. Sustainability terms included “energy”, “life cycle”, “environmental impact”, “recycling”, “recycled filament”, “bio-based”, “natural fibre”, “circular economy”, “repair”, and “techno-economic”. Application blocks included “pallet”, “logistics”, “load-bearing polymer structure”, “membrane feed spacer”, “spacer-filled channel”, “pressure drop”, “mass transfer”, “fouling”, and “cleaning”. Studies were included when they provided direct application evidence or clearly transferable evidence on process physics, material behaviour, sustainability, or qualification. Studies were excluded when they were duplicate reports, nontechnical product pages, or lacked a clear connection to polymer AM, the two application classes, or lifecycle interpretation.

For synthesis, the retained literature is interpreted in three evidence classes. Direct application evidence covers additively manufactured feed spacers, membrane-related flow components, pallet logistics requirements, and any component-level studies that directly reproduce the intended service function. Enabling evidence includes SLA, DLP, FDM, large-format extrusion, lattices, TPMS architectures, process parameters, nanocomposites, natural-fibre systems, ceramics, and recycled feedstocks that inform application design without testing the final component. Cross-cutting evidence includes life-cycle assessment, circularity, Industry 4.0, process monitoring, machine learning, and techno-economic analysis. This hierarchy is used throughout the paper so that a measured result is not conflated with an extrapolated design opportunity.

The analytical framework uses three linked levels. The manufacturing-process level covers energy demand, build rate, dimensional capability, support requirements, post-processing, emissions, and material utilization. The component level covers as-printed geometry, mechanical or hydrodynamic function, durability, cleaning or maintenance, and manufacturability. The lifecycle level covers feedstock origin, transport, service-life benefit, repair, reuse, recycling, and disposal. A sustainability claim is considered strong only when improvement at one level is not achieved through a disproportionate burden at another [1,2,3,4,5,10].

This framework also separates manufacturing-stage efficiency from use-stage efficiency and intrinsic material origin from system-level performance. A spacer may require more energy to print than a commercial mesh yet still reduce lifecycle burden if it measurably lowers pumping demand, fouling, or cleaning over a sufficiently long service period. A pallet-scale component may justify a higher-energy manufacturing route only if mass reduction, damage resistance, repair, or material recovery produces a persistent service benefit. Likewise, a bio-based resin or recycled filament is not automatically sustainable when reduced durability causes early replacement. Material origin, property retention, process yield, service life, and end-of-life treatment are therefore treated as coupled variables [6,7,8,9,10,39,40,41,42,43,44,45].

**Figure 2:**
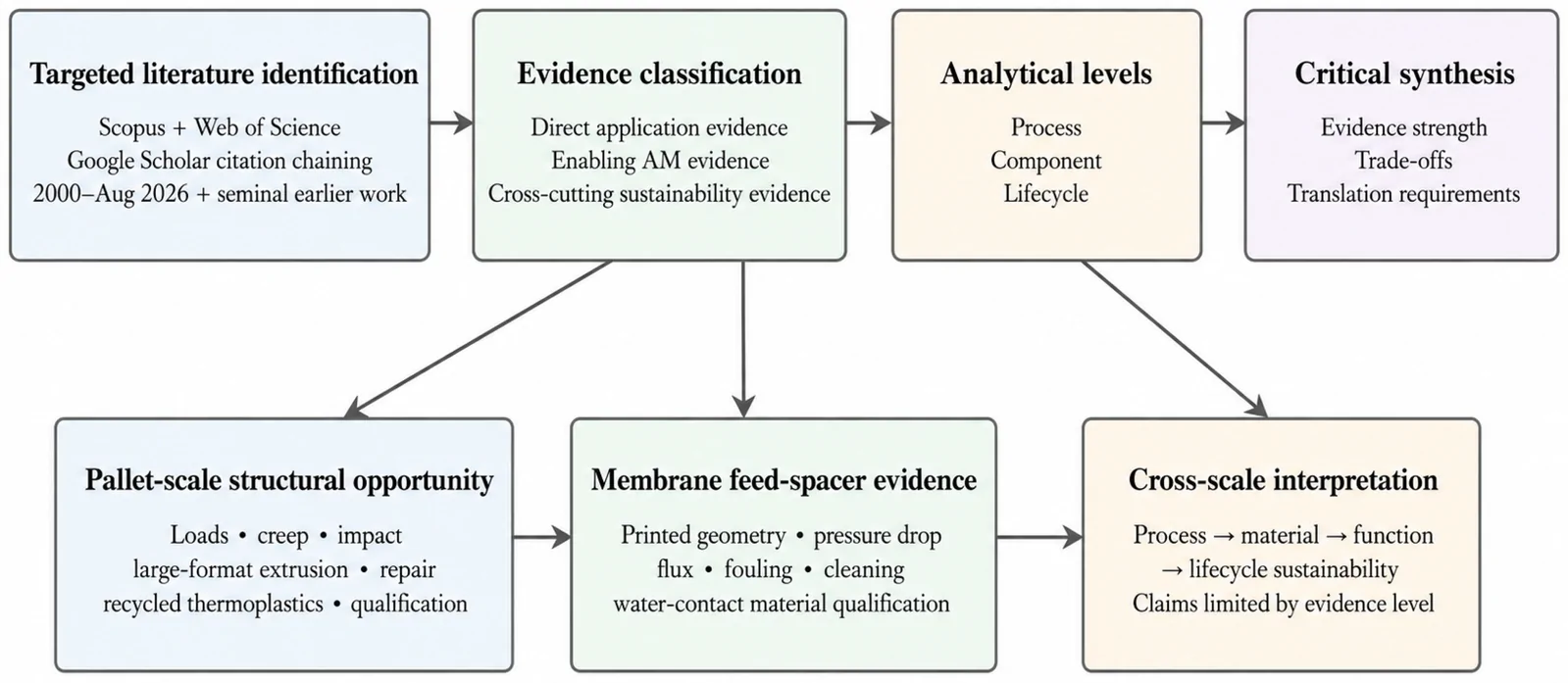
Analytical framework for the critical integrative review, linking targeted literature identification to evidence classification and process-, component-, and lifecycle-level synthesis.

Four questions guide the review: (i) which AM process characteristics are most relevant to pallet-scale structural applications and feed-spacer design; (ii) how do material, geometry, and process parameters interact with application performance; (iii) under what conditions can use-phase and circularity benefits outweigh manufacturing burdens; and (iv) what evidence is still required for industrial qualification and scale-up? Figure 2 now presents the analytical framework rather than a PRISMA-style screening flow. PRISMA [46] was not applied because the review was not prospectively designed as a systematic review. The original iterative search did not preserve reliable stage-by-stage database hit counts, and such counts are therefore not retrospectively fabricated. The revised cited evidence base contains 144 sources, with direct, enabling, and cross-cutting evidence identified explicitly in the synthesis. Table 1 compares the three principal polymer AM routes used throughout the review.

## 3. Sustainable Additive Manufacturing: Process–Material–Function Framework

### 3.1. Design Freedom, Digital Manufacturing, and Resource Efficiency

AM is often described as a material-saving technology because it builds parts layer by layer. That description is directionally correct but incomplete. The deeper advantage is that manufacturing constraints can be moved upstream into computational design. Topology optimization, lattice structures, triply periodic minimal surfaces (TPMS), graded density, internal channels, and part consolidation allow engineers to distribute material according to local functional demand. In this sense, AM shifts sustainability from a process-efficiency problem toward a design-efficiency problem. The environmental gain can become substantial when the redesigned component is lighter, performs better, lasts longer, or eliminates assembly and maintenance operations [4,26,27,28,29,34,47,48,49,50].

**Figure 3:**
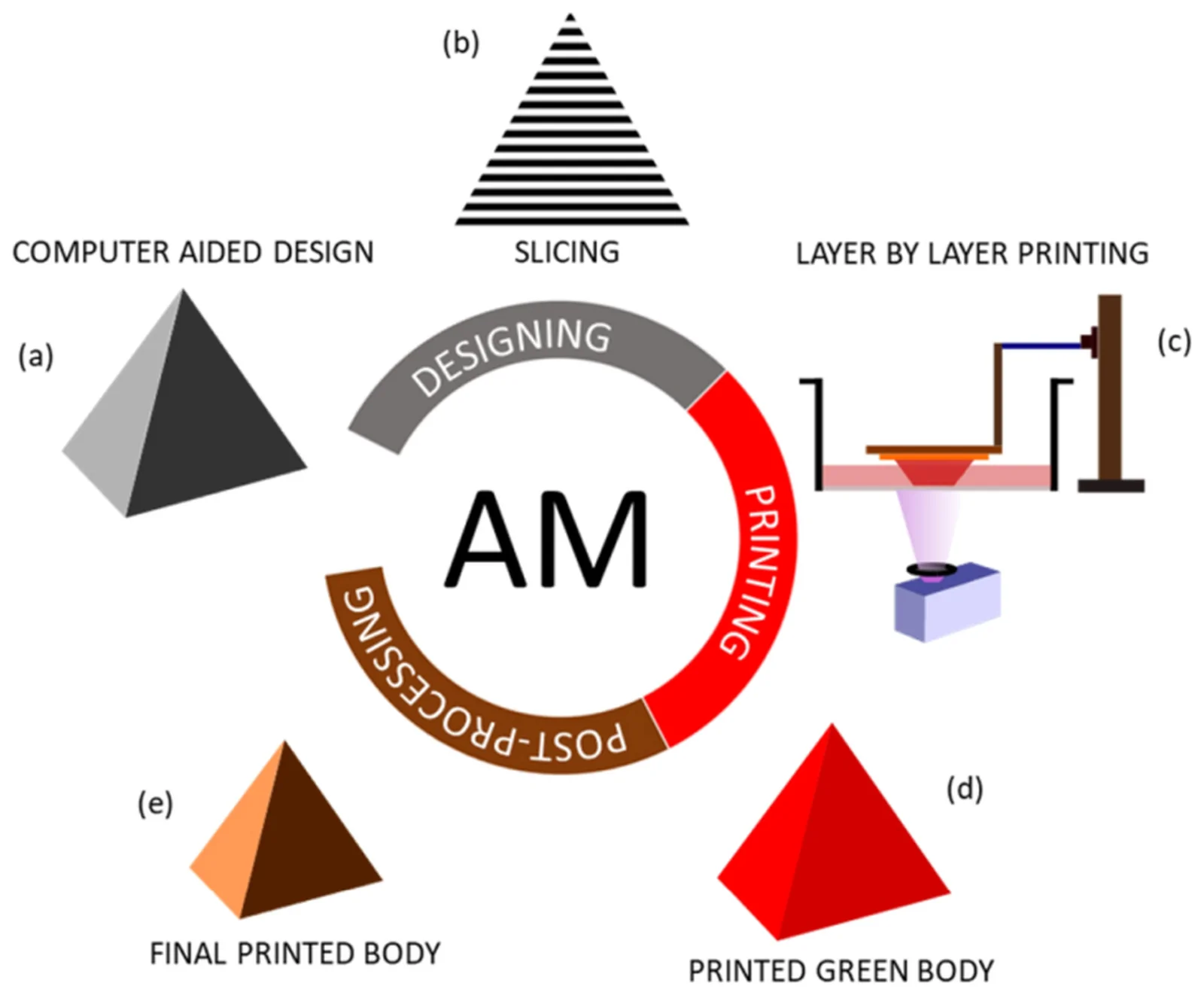
Step-by-step manufacturing process in AM: CAD modelling, slicing, layer-wise printing, green-part formation, and post-processing. Adapted from Ref. [18].

Figure 3 illustrates the digital-to-physical workflow common to AM: CAD modelling, slicing, layer-wise fabrication, and post-processing. Each stage contains sustainability decisions. CAD determines whether geometry genuinely uses AM freedom. Slicing sets layer height, infill, support, path planning, exposure, and deposition strategy, thereby affecting build time and energy. Printing determines actual material and electricity use, while post-processing can add solvents, thermal curing, machining, drying, or cleaning. Process variables therefore influence sustainability through both resource consumption and part quality: a parameter change that shortens build time but increases rejection, warpage, poor bonding, or rework may not lower lifecycle impact [6,8,18,51,52,53].

The digital nature of AM also supports distributed production and digital inventory. Instead of storing large inventories of physical spares, a validated digital design can be manufactured near the point of need, provided that process capability and material traceability are controlled. This concept aligns with Logistics 4.0 and wider Industry 4.0 integration, in which connected manufacturing, data-rich process control, and flexible supply networks are used to reduce delays and improve responsiveness [5,11,12]. For pallets, this could support localized replacement of damaged modules or production near logistics hubs. For membrane components, it could support rapid geometry customization for research, pilot-scale systems, or specialized treatment requirements.

However, digital distribution introduces a quality challenge. A CAD file is not equivalent to a qualified physical product. Printer architecture, thermal history, resin age, filament moisture, layer settings, calibration, and post-processing can change final properties. Sustainable distributed manufacturing, therefore, requires validated process windows, material specifications, quality-control protocols, and a clear relationship between digital design intent and measured component performance. These issues are especially important for structural pallets, where load safety is critical, and for water-treatment spacers, where dimensional deviations may alter pressure drop and flow distribution.

**Figure 4:**
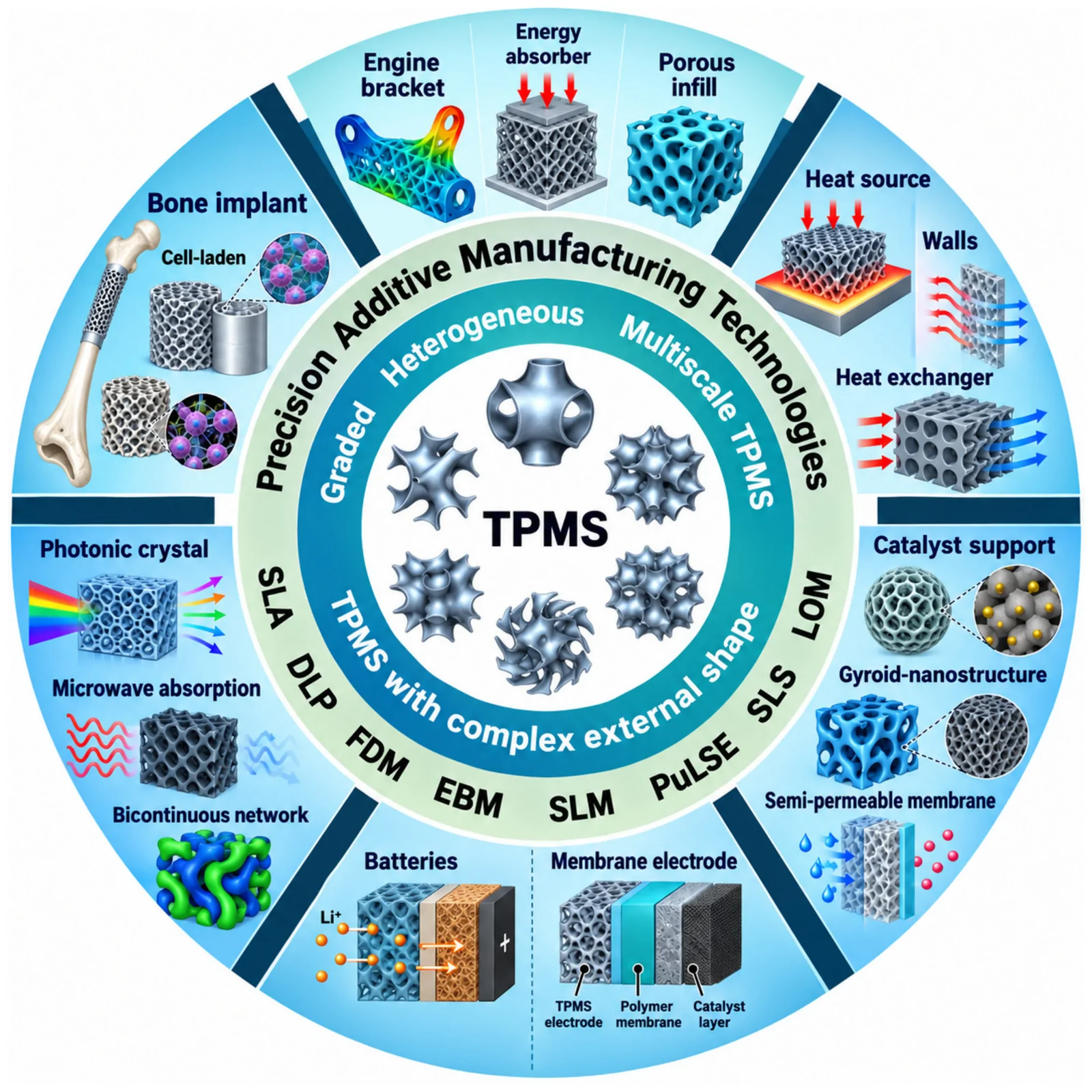
Precision additive manufacturing technologies from solid structures to lattice and TPMS architectures. Adapted from Ref. [48].

The transition from solid to cellular and TPMS-inspired architectures, illustrated in Figure 4, represents one of the most important design opportunities created by AM. Cellular structures provide a way to decouple external geometry from internal material distribution. For pallets, this allows local stiffness to be increased near deck interfaces, legs, runners, or fork-entry regions while removing material from low-stress zones. For spacers, periodic and smooth-surface architectures provide a means to control flow pathways and local mixing. The same mathematical design language can therefore support two different sustainability mechanisms: structural lightweighting in logistics and hydrodynamic optimization in membrane processing [48].

### 3.2. Vat Photopolymerization: SLA and DLP

SLA is one of the earliest vat-photopolymerization processes and remains a benchmark for high-resolution polymer AM. A focused ultraviolet source scans a defined path to polymerize selected regions of a liquid resin. Successive layers create a three-dimensional structure with fine feature definition and comparatively smooth surfaces. These characteristics make SLA attractive for intricate flow devices, microfluidic structures, model components, and geometries in which surface quality and dimensional fidelity are more important than raw deposition rate [19,21,54,55,56,57].

The point-scanning mechanism has direct implications for sustainability. High geometric accuracy can reduce the need for machining and can enable thin walls or fine features that minimize material use. At the same time, large projected areas may require long exposure paths, while support structures, resin handling, cleaning, and post-curing add process steps. Sustainability therefore depends on whether the precision of SLA is functionally necessary. For a full-size pallet, the scale and build-time burden can be difficult to justify. For a small, hydrodynamically optimized feed spacer or a research-scale flow insert, the same precision may create substantial value because geometric detail directly controls performance [6,19].

Photopolymer networks are formed through light-initiated cross-linking, and their mechanical performance depends on resin chemistry, conversion, exposure, layer orientation, and post-cure history. Printed parts can exhibit anisotropy because interlayer interfaces and cure depth differ from the bulk polymer network. Orientation effects reported for 3D-printed resins reinforce the need to explicitly evaluate loading direction rather than rely on datasheet properties [20]. For structural applications, this means coupon-level tensile data should be supplemented by bending, impact, creep, and component-level loading in the actual print orientation.

Post-curing can increase conversion and stabilize properties, but excessive exposure may also increase brittleness or dimensional change depending on chemistry. From a sustainability perspective, post-curing is a trade-off: it adds energy consumption, yet it can extend service life and reduce premature failure. A lifecycle interpretation is therefore necessary. A slightly higher manufacturing energy burden may be justified if post-curing materially improves durability, chemical resistance, or water stability [6,21].

One response to the brittleness and limited thermal performance of conventional SLA resins is the incorporation of nano- and micro-scale reinforcement. Metal–metal-oxide nanoparticles have been shown to improve mechanical and thermal properties when dispersion and interfacial bonding are adequately controlled [22]. Silica-containing systems and other particle-reinforced photopolymers similarly demonstrate that vat-photopolymerization can move beyond simple prototyping resins toward engineered composites [58]. The central processing difficulty is that reinforcement changes resin viscosity, optical penetration, sedimentation behaviour, and cure kinetics.

These interactions are particularly relevant to sustainable design because adding reinforcement is beneficial only if it improves functional life without compromising print reliability or recyclability. Agglomerates can act as defects, increase scattering, and reduce resolution. High particle loading can complicate resin recovery and end-of-life treatment. Therefore, the most sustainable reinforcement strategy is not necessarily the formulation with the highest filler fraction; it is the formulation that achieves the required stiffness, toughness, wear resistance, or chemical stability with the lowest complexity and stable printability [22,58].

Sustainability-focused SLA research increasingly targets renewable monomers, lignin-derived systems, cellulose-containing materials, and lower-toxicity formulations [6,40,41]. Wood-reinforced photopolymer composites show that lignocellulosic fillers can be incorporated into stereolithographic systems, although optical absorption, particle settling, and interfacial bonding remain important constraints [41]. More recent work extends this direction: an acrylated avocado-oil resin containing lignin-based nanocontainers was successfully printed by LCD-SLA and showed compost biodegradation, while a separate techno-economic/LCA study of a 10 wt% lignin-blended resin reported a production cost of $38.43 kg^−1^ and a global-warming impact of 6.64 kg CO_2_-eq kg^−1^ resin [59,60]. These examples show that renewable content can be quantified and engineered, but it does not remove the need to evaluate cure, durability, post-processing, and end-of-life behaviour.

Bio-derived content should not be equated automatically with circularity. Cross-linked thermoset networks are intrinsically more difficult to remelt than thermoplastics, and many vat-photopolymerization systems remain challenging to recycle after full cure. Consequently, sustainability strategies for SLA should include resin minimization, recovery of unused resin, longer service life, low-toxicity chemistry, reduced solvent demand, and, where possible, chemically recyclable or reprocessable network design. In pallet applications, this limitation weighs against SLA for high-volume, large parts. In spacer applications, where component mass is relatively small and functional gains can be substantial, high-resolution resin printing may still be justified if leachability and durability are carefully validated [61].

DLP belongs to the same vat-photopolymerization family as SLA but cures an entire two-dimensional layer using projected light. Because layer exposure is area-based rather than traced point by point, the time required for each layer is less sensitive to the number of discrete features within it. This gives DLP an important throughput advantage for arrays of small parts or intricate repeated structures. At the same time, in-plane resolution is linked to projected pixel size and optical characteristics, while cure depth remains governed by resin absorption and exposure conditions [18,24,25].

These process attributes are well aligned with feed-spacer research because repeated filaments, nodes, and channels benefit from whole-layer exposure. For pallet-scale structures, however, projection area, resin volume, part mass, and post-processing become increasingly restrictive. DLP is therefore best viewed as a high-resolution, high-feature-density process within a finite build envelope rather than a universal replacement for large-format thermoplastic extrusion [18,24,25,62].

DLP performance depends strongly on the resin optical response. Exposure time, light intensity, layer thickness, photoinitiator concentration, absorber content, viscosity, and filler loading determine cure depth and lateral overcure. Under-exposure can create weak or incompletely cured layers, whereas over-exposure can broaden features and reduce dimensional fidelity. Parameter-optimization studies therefore show that dimensional accuracy, degree of cure, surface quality, and build time must be optimized together rather than independently [18,25,62].

For feed spacers, small dimensional errors can become hydrodynamically significant because filament diameter, gap size, crossing angle, and surface roughness influence flow area and local shear. Consequently, process calibration is not merely a geometric quality issue but a functional requirement. A nominally identical CAD spacer printed with different exposure settings can produce a different pressure-drop response. This sensitivity strengthens the case for reporting measured dimensions, surface characteristics, and actual open-channel geometry alongside nominal design values.

Photopolymerization also faces oxygen inhibition, shrinkage, residual monomer, and post-cure dependence. In water-treatment applications, incomplete cure is particularly important because leachable species can alter water quality or biological fouling. Thorough post-curing and extraction may improve stability but add time, solvent, and energy. A sustainable DLP workflow should therefore minimize residual chemistry and post-processing burden while still meeting dimensional, leachability, and durability requirements [6,13,14,15,16].

DLP has expanded well beyond conventional acrylate resins. Ceramic-filled formulations can be printed, then debound and sintered to form dense zirconia or other ceramic structures [19,21,22]. Integrated ceramic membranes produced by DLP demonstrate the relevance of this route to separation technologies [63]. These developments broaden the material space for chemically aggressive or high-temperature environments, but they also introduce shrinkage, debinding energy, sintering energy, particle sedimentation, and dimensional-compensation challenges.

Cellulose-based hydrogels and other more sustainable photopolymer systems show a different direction: replacing some petroleum-derived chemistry with bio-based constituents while preserving digital light processing [43]. Such materials are highly relevant conceptually because they demonstrate that sustainability can be addressed through chemistry as well as geometry. Their direct suitability for long-term membrane service depends on swelling behaviour, mechanical stability, microbial interactions, and chemical resistance, so material sustainability must remain subordinate to application suitability.

DLP can reduce exposure time per layer compared with point-scanning SLA, but the environmental inventory still includes resin production, machine standby, optical projection, recoating, support material, washing, post-curing, rejected builds, and end-of-life treatment [6,18]. A recent controlled energy study comparing conventional layer-wise DLP with volumetric photopolymerization found that the volumetric route used approximately one-half to one-sixth of the DLP energy and one-ninth to one-twenty-second of the DLP manufacturing time for the tested bilayer structures [64]. The value of this result is not a universal ranking of processes; rather, it demonstrates how strongly layer-wise exposure strategy and system boundary can affect the energy result.

For industrial scale-up, enlarging the DLP build area can reduce effective pixel resolution unless the optical architecture is redesigned. Multiple projection systems or tiled exposures can increase area but introduce calibration complexity, while large cross-sections increase peel, separation, and shrinkage forces. These factors make DLP compelling for small and medium repeated-spacer geometries but less credible for whole pallet-scale structures. More realistic structural roles include high-detail inserts, joints, sensor housings, fixtures, or prototype validation [18,24,25].

From a circularity perspective, uncured resin can often be filtered and reused within controlled limits, but fully cured thermoset parts are more difficult to recycle than thermoplastic FDM components. DLP sustainability research should therefore prioritize low-toxicity formulations, renewable monomers, solvent reduction, high build yield, long service life, and chemical recycling where feasible. These priorities are consistent with wider efforts to make SLA/DLP materials and processes more sustainable [65].

### 3.3. Material Extrusion: FDM, Structural Scalability, and Thermoplastic Circularity

FDM, also known as fused filament fabrication, deposits a thermoplastic bead through a heated nozzle according to a programmed toolpath. Its simplicity of equipment, broad material availability, relatively low capital cost, and compatibility with open-source slicing have made it one of the most widely accessible AM processes. For sustainable manufacturing, its most important distinguishing feature is the use of melt-processable thermoplastics, which can, in principle, be re-extruded, repaired, welded, or mechanically recycled more readily than permanently cross-linked photopolymers [44,45,66,67].

FDM is the most immediately plausible of the three reviewed processes for pallet-scale thermoplastic structures. Large-format extrusion and pellet-fed systems can reduce desktop build-envelope constraints and raise deposition rate. Scale, however, introduces thermal gradients, warpage, shrinkage, weak inter-bead interfaces, surface waviness, and long uninterrupted builds. A pallet-scale structure cannot therefore be justified by scaling a desktop coupon; geometry, bead size, path planning, local reinforcement, service load, build yield, and machine utilization must be treated together [68,69,70,71,72,73,74,75,76,77,78].

Layer thickness, raster angle, infill density, nozzle temperature, bed temperature, print speed, cooling rate, build orientation, contour strategy, and air gap simultaneously affect mechanical response, dimensional quality, material use, and energy. Higher infill can increase stiffness but also raises material consumption and build time. Smaller layer height can improve surface quality and interlayer contact but prolongs machine operation. Higher nozzle or chamber temperature can improve fusion while increasing thermal load and, in some polymers, degradation or emissions. Recent sustainability reviews therefore treat FDM parameter selection as a multi-objective problem rather than a mechanical-strength optimization alone [51,52,53,73,78,79,80,81,82,83].

Anisotropy is especially important for structural design. Material-extrusion components are usually strongest along continuous deposited roads and weaker across layer interfaces. This directional behaviour has been demonstrated for unfilled and composite FDM systems [70,71,75,84]. For pallet-scale design, load paths should be aligned with strong deposition directions where practical, and critical regions should not depend solely on interlayer tensile strength. Finite-element models should therefore use process-informed anisotropic properties rather than isotropic bulk polymer datasheet values.

Process optimization should therefore be multi-objective. Maximizing mechanical strength alone can produce unnecessarily dense, slow, and energy-intensive parts. More appropriate objectives combine load capacity, mass, build time, energy, dimensional accuracy, and material cost. Multi-objective optimization and design-of-experiments approaches are particularly relevant because FDM contains many interacting variables [80,81,82,83,85]. For sustainable pallets, the optimum process window is likely to be the lowest material and energy combination that meets all defined structural and durability constraints, not the setting that maximizes a single coupon property.

Composite filaments containing short fibres, mineral fillers, or functional particles can substantially extend the stiffness, thermal resistance, wear behaviour, or dimensional stability of FDM parts [66,67]. This is attractive for pallet applications where conventional PLA or ABS may not provide adequate creep resistance, heat resistance, or durability under repeated loads. Engineering thermoplastics and reinforced grades can enable thinner structural sections and greater reuse, but they often require higher processing temperature, controlled drying, heated chambers, and more demanding equipment.

The sustainability benefit of reinforcement is therefore conditional. A reinforced material that doubles service life or permits major mass reduction can lower lifecycle impact even if its production energy is higher. Conversely, fibre-filled polymers can be harder to mechanically recycle and may cause nozzle wear or reduced interlayer fusion. The design task is to identify where reinforcement is functionally necessary and where unfilled or recycled material is sufficient. Graded or localized reinforcement is conceptually attractive because it places premium material only in high-demand regions, although practical multi-material deposition and recycling remain challenges [66,86].

Recycled thermoplastic feedstock is one of the strongest circularity arguments for FDM, but the mechanical penalty is polymer- and process-dependent. Pinho et al. reported approximately 33% reductions in tensile stress and flexural strength for post-consumer recycled PLA, whereas recycled ABS in the same study showed no meaningful mechanical-property loss [87]. Olawumi et al. reported retention of up to 90% of original tensile strength after one recycling cycle and about 80% after three cycles, together with 30% lower recycling-process energy and a 25% lower carbon footprint under the conditions studied [9]. A 2025 post-consumer PLA study further showed that 100% recycled PLA reduced tensile and flexural strength by 48.4% and 49%, while blends containing up to 50% recycled PLA retained more than 90% of virgin-PLA flexural performance [88]. These studies do not support one universal recycled-material penalty; they support feedstock-specific qualification and controlled blending.

For pallets, a closed-loop system is conceptually attractive because damaged components are relatively large and material recovery can be organized at logistics hubs. A circular pallet system could combine modular design, repair, replaceable high-wear zones, mechanical recycling, and controlled blending of recycled with virgin polymer where needed. The critical requirement is material traceability. Unknown mixtures, contamination, moisture, and uncontrolled degradation can create unacceptable structural variability. Circularity therefore requires quality-control specifications for recycled feedstock rather than simply the presence of recycled content [9].

For feed spacers, recycled FDM materials are possible but chemical stability, surface cleanliness, and leachability become more stringent. Recycled feedstock may contain additives, pigments, degradation products, or contaminants that are acceptable in a pallet but unsuitable in contact with process water. Application-specific qualification is therefore essential. This contrast illustrates why circular economy strategies must be matched to service environment rather than transferred uncritically between products.

FDM machines consume energy through nozzle heating, bed heating, chamber conditioning, motors, electronics, and auxiliaries. Reported desktop FFF volumetric specific energy consumption spans 24.8–85.7 kJ cm^−3^, showing that machine configuration and process settings can change the energy intensity substantially [51]. Lunetto et al. likewise showed that specific energy consumption decreases as useful deposition rate increases and reported 9.47 MJ (2.63 kWh) for a 149.5 g validation build, corresponding to a modelled 66.2 MJ kg^−1^ (18.4 kWh kg^−1^) under that study’s conditions [52]. Energy-efficient parameter selection is therefore legitimate, but faster deposition is sustainable only if dimensional accuracy, interlayer integrity, and failure rate remain acceptable [8,51,52,53,89].

Material extrusion can also release ultrafine particles and volatile organic compounds, with emissions depending on polymer chemistry, temperature, ventilation, and machine enclosure. Sustainable implementation should therefore include occupational exposure alongside energy, material waste, and cost. A parameter setting that reduces print time but increases emissions or reject rate can simply shift the burden rather than remove it [8,53].

### 3.4. Advanced Feedstocks, Multifunctionality, and Hybrid Systems

Photopolymer selection for SLA and DLP is governed by different constraints. Resin viscosity must permit recoating; optical absorption must allow controlled cure depth; photoinitiator chemistry must match the light source; and the cured network must provide adequate stiffness, toughness, dimensional stability, and environmental resistance. Increasing cross-link density can improve modulus and thermal stability but may also increase brittleness. Introducing flexible segments can improve toughness but may reduce heat resistance or dimensional precision [23,90].

Composite AM is attractive because it allows material functionality to be tailored without changing the basic manufacturing platform. Short fibres can increase stiffness; silica or ceramic particles can improve dimensional or thermal stability; magnetic particles can introduce actuation; conductive fillers can support electrical functionality; and bio-derived fillers can reduce fossil-derived content [22,41,42,58,66,91]. Yet fillers affect more than the final property. They change viscosity, flow, cure depth, nozzle wear, sedimentation, interfacial adhesion, and sometimes the recyclability of the matrix [92].

Natural reinforcements deserve separate treatment because they can lower fossil-derived reinforcement content while introducing new process constraints. Continuous flax/PLA has demonstrated structural potential, and recent reviews cover continuous and pelletized natural-fibre systems across automotive, aerospace, and consumer applications [32,92,93]. Their sustainability case depends on moisture control, fibre variability, interface quality, nozzle or feeding reliability, anisotropy, and whether fibre–matrix separation is feasible at end of life. Natural origin alone does not compensate for poor print yield or premature failure.

These sectoral examples also illustrate why “real-world” AM sustainability should be reported with component-level metrics. A lightweight aerospace bracket, customized medical device, tooling insert, automotive fixture, sensor housing, logistics module, or membrane spacer may each justify AM for a different reason. The relevant evidence may therefore be mass saved per service life, avoided tooling, shorter supply chain, improved patient fit, reduced assembly, lower pumping energy, or longer cleaning interval. The functional unit must follow the service provided rather than the printer used [26,27,28,29,30,31,32].

A sustainable composite formulation should therefore be judged by property gained per unit of added complexity. If a small filler fraction meaningfully increases service life, reduces section thickness, or prevents failure, the lifecycle benefit may be substantial. If the same filler causes agglomeration, print defects, poor interlayer bonding, or difficulty in recovery, the nominal material improvement can be offset by higher rejection rates and end-of-life burden. This is why dispersion quality and interfacial bonding are as important as filler type.

Multifunctionality is especially promising when it replaces separate components. A conductive or magnetic feature printed into a pallet insert could support sensing or identification, while a functional spacer surface could alter wetting, adsorption, or fouling behaviour. The strongest sustainability case occurs when added functionality reduces assembly, maintenance, or operating energy. Conversely, embedding incompatible materials permanently can undermine circularity. Future designs should therefore favour separable modules or compatible material families when introducing multifunctional features.

Ceramic SLA/DLP expands AM into chemically stable and high-temperature materials by printing a particle-loaded photopolymer followed by debinding and sintering [24,61,63,94]. For water-treatment components, this pathway can offer chemical resistance and precise porous architecture. The trade-off is the substantial thermal processing required after printing, together with shrinkage and the risk of cracking or distortion. The environmental advantage is therefore not obvious and must be evaluated against service life and performance.

**Figure 5:**
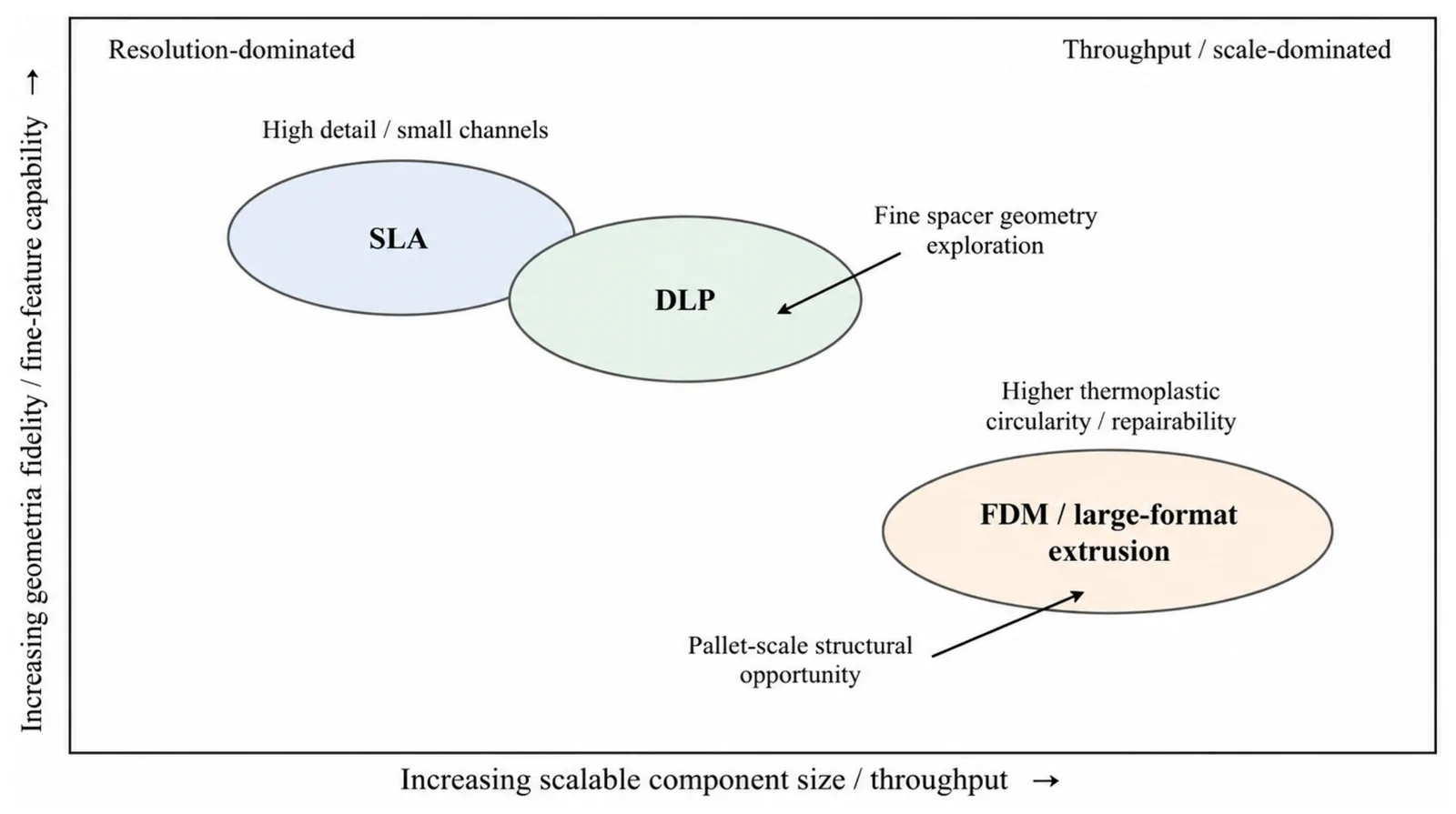
Qualitative process-capability map for SLA, DLP, and FDM/large-format extrusion. Axis positions indicate relative trends rather than measured numerical rankings.

Hybrid systems may offer a more efficient route than monolithic AM. A conventional or extruded pallet shell could incorporate AM-optimized joints, impact absorbers, or sensor housings, while a membrane module could combine a conventionally manufactured membrane with AM spacers or local flow distributors. Hybridization concentrates AM where geometric freedom creates measurable value and retains high-throughput processes for simple regions. Figure 5 therefore presents a qualitative process-capability map, without unsupported numerical axes, showing why vat photopolymerization aligns more closely with fine spacer geometries and why FDM/large-format extrusion aligns more closely with pallet-scale structural opportunities. Table 1 compares SLA, DLP, and FDM in terms of mechanism, dimensional/detail capability, sustainability strengths, limitations, and application fit [4,18,25,26,27,28,29,62,68].

Across these process families, the central design principle is process-application matching. High resolution is valuable only when it controls functional performance, and high deposition rate is valuable only when the resulting geometry and interlayer integrity remain adequate. Sustainable AM, therefore, requires the simultaneous consideration of feature scale, material chemistry, build rate, post-processing, durability, and recovery, rather than ranking technologies by a single manufacturing metric.

## 4. Pallet-Scale Structural Opportunities: Evidence, Extrapolation, and Qualification Needs

### 4.1. Service Requirements and Evidence Status

Transportation pallets appear geometrically simple, but their service combines global bending, local indentation, fork-entry impact, racking, deck loading, conveyor contact, repeated handling, environmental exposure, and long-term creep. Conventional wood and moulded-plastic pallets also benefit from mature supply chains, repair practices, standards, and established economics. Any AM route must therefore compete on total service value rather than on printability or mass alone.

A critical limitation of the present evidence base is that the cited literature does not provide a primary peer-reviewed study demonstrating a fully additively manufactured transportation pallet subjected to complete pallet-level qualification. Reference [12] concerns sustainable palletizing/warehouse operations, while [95] reports 3D-printed devices for vibration mitigation during fruit-box transport rather than a printed pallet. The pallet discussion in this review is therefore intentionally framed as a translation analysis built from large-format polymer AM, structural FDM, cellular-architecture, repair, recycling, and logistics evidence [11,12,31,34,66,67,68,69,70,71,72,73,74,75,76,77,78,84,89,95,96,97,98,99,100,101,102,103,104,105,106,107,108]. The proposed benefits below are design opportunities and qualification hypotheses unless direct component evidence is stated.

Within that evidence boundary, the most credible sustainability mechanisms are selective rather than monolithic: topology optimization and cellular architecture can reduce material in low-demand zones; large-format thermoplastic extrusion can increase structural scale; local reinforcement can protect fork-entry or support regions; modular design can enable replacement of damaged zones; and known-material take-back streams can support controlled recycling. The relevant question is therefore not whether a conventional pallet geometry can be printed, but which pallet functions obtain enough structural, maintenance, logistics, or circularity value from AM to justify its energy, time, and qualification burden.

### 4.2. Material Selection and Structural Architecture

Pallet materials must balance stiffness, toughness, creep resistance, impact tolerance, temperature capability, moisture resistance, repairability, cost, and recyclability. FDM literature on PLA, ABS, polymer blends, composites, and high-performance thermoplastics shows that mechanical response varies substantially with material and process settings [67,80]. PLA can provide good stiffness and ease of printing, but may be limited by heat resistance and brittle behaviour in some environments. ABS provides toughness and thermal capability but requires thermal management and emits more volatile species during printing. Reinforced and engineering thermoplastics can improve structural performance but may increase cost, processing energy, and recycling complexity.

The correct comparison should therefore be performed on a functional basis, such as load capacity per unit mass, cycles to failure, stiffness retention after environmental conditioning, or service life per kilogram of material. This functional-unit approach is consistent with lifecycle-based sustainability assessment because it prevents a low-mass but short-lived design from being misclassified as sustainable [10].

Recycled polymer offers additional value when properties remain sufficiently stable. Pallet systems are particularly suitable for controlled take-back because the product is already part of a logistics network. A manufacturer or user can potentially collect damaged pallets, identify material grade, reprocess suitable fractions, and produce replacement components. Such a system is more credible than generic post-consumer recycling because material history can be better controlled [7,9,44,45].

AM enables the separation of the external pallet envelope from its internal structural architecture. Instead of a uniformly solid deck or beam, the designer can use ribs, honeycombs, trusses, graded lattice density, or TPMS-inspired cores. The objective is to transmit loads through efficient paths while preserving local crush resistance at contact points. Cellular architecture can also provide damping, drainage, and impact energy absorption when appropriately designed [96,97,98,99,100,101,102,103,104,105,106].

Not all lattice geometries are advantageous. Very fine cells can increase print time and create inaccessible voids that trap contamination or are difficult to inspect. Extremely low relative density can lead to local buckling, while unsupported internal features can result in poor deposition quality. For pallets used in food, pharmaceutical, or hygienic logistics, cleanability may also outweigh maximum mass reduction. Thus, topology optimization should include manufacturing and sanitation constraints, not merely stiffness or mass constraints.

A promising approach is hierarchical design. The pallet can be divided into functionally distinct zones: broad deck regions optimized for bending stiffness, fork-entry regions reinforced for local impact and indentation, feet or runners optimized for compressive stability, and sacrificial or replaceable edge regions designed for damage tolerance. This type of zoned architecture uses AM, where it adds value and can simplify repair by allowing high-wear modules to be replaced rather than discarding the entire pallet.

Thermoplastic selection for FDM should begin from the service envelope rather than printer availability. A pallet material experiences sustained and cyclic bending, local compression, impact, abrasion, humidity, temperature variation, and potentially chemical exposure. The relevant property set therefore extends beyond room-temperature tensile strength to include modulus, strain-to-failure, impact resistance, creep compliance, fatigue behaviour, heat-deflection response, moisture sensitivity, and weldability or repairability. Reviews of FDM materials show that these properties are strongly coupled to processing history, so a nominal polymer grade cannot be treated as a complete material definition [67].

PLA is attractive because of its printability, stiffness, and the possibility of using bio-derived feedstock, but its thermal and long-duration load response can limit its use in demanding logistics environments. ABS offers greater toughness and temperature resistance in many contexts, but requires stronger thermal management during printing and is associated with higher volatile emissions. Polyamides can provide toughness and wear resistance, yet moisture uptake changes dimensions and mechanical properties. Higher-performance thermoplastics can extend temperature and creep capability, but they require heated build environments and can increase process energy. Material sustainability, therefore, cannot be ranked independently of the operating environment.

For large load-bearing structures, the designer should also distinguish between material stiffness and structural stiffness. A relatively modest polymer can perform effectively when the architecture places material along load paths, while an intrinsically stronger polymer may be wasted if the geometry is inefficient. This interaction is precisely where AM can create sustainability gains. Material selection and topology optimization should be undertaken together so that high-performance or virgin material is used only where the structure requires it, with recycled or lower-impact material used elsewhere when multi-material recovery remains feasible.

Long-term creep deserves particular emphasis. Thermoplastics can continue to deform under sustained stress at loads far below their short-term failure strength. A pallet stored under rack loading for hours or days may therefore experience deformation that is not predicted by a standard tensile coupon. Creep is also temperature-dependent. Future pallet studies should characterize time-dependent behaviour in the relevant print orientation and then incorporate viscoelastic or viscoplastic response into structural simulation. This is necessary to prevent lightweight designs from achieving short-term strength at the expense of serviceability.

### 4.3. Print Orientation, Joining, Structural Validation, and Reliability

Large pallets may exceed the build envelope of many printers, making segmentation and joining unavoidable. Segmentation introduces interfaces that can become structural weak points. Mechanical locking, dovetail joints, polymer welding, fasteners, or adhesive bonding can be used, but each approach affects disassembly and recycling. Design for AM should therefore treat joining as part of the structural architecture rather than an afterthought.

Print orientation should be selected with the dominant stress field in mind. Interlayer tensile loading should be minimized in critical regions because FDM anisotropy can reduce strength normal to the deposited layers [84]. Continuous contours and raster paths should align with principal load directions where practical. Local infill changes, additional perimeters, thicker beads, or embedded reinforcement can strengthen high-demand regions without uniformly increasing mass.

Component validation should extend beyond static ultimate load. Repeated forklift handling, drop or impact events, creep under stored loads, rack support, temperature variation, humidity, and surface wear can govern real-world life. Failure modes should be documented because a design that passes one static test may still fail due to interlayer delamination, creep, notch cracking, or local crushing. Standardized, application-representative test protocols are therefore a major requirement for translating AM pallet concepts into deployable logistics products.

Pallet qualification should follow a hierarchy from material coupons to structural subcomponents and finally to representative pallet-level tests. Coupon testing is useful for establishing directional elastic and failure properties, but it cannot reproduce stress concentrations around fork openings, deck interfaces, joints, or support points. Subcomponent tests can isolate these local mechanisms, while full-structure tests reveal load redistribution and system-level failure.

At the material level, tensile, compressive, flexural, and impact responses should be measured in relevant print orientations. Interlayer fracture or notched testing may be justified when delamination is a likely failure mode. For thermoplastics, creep and temperature dependence should be included. For reinforced materials, the effect of fibre orientation and process-induced voids should be documented [66,80,81,82,84].

At the component level, tests should reproduce floor support, rack support, forklift or tine contact, local deck loading, impact, and repeated handling. Deflection should be measured in addition to ultimate load because excessive deformation can cause service failure before material fracture. Cyclic tests should be designed around realistic load ranges and should document stiffness loss, permanent set, crack growth, or interlayer separation. Environmental conditioning should be included where temperature, humidity, water, or chemicals are relevant.

Correlation between experiment and simulation is particularly valuable. Finite-element models can evaluate many geometric variants, but they should be calibrated using process-specific material properties and validated against measured structural response. Isotropic properties taken from bulk material datasheets can lead to misleading conclusions for FDM. A validated simulation can then be used for topology optimization, local reinforcement, and sensitivity analysis without requiring every design iteration to be printed.

### 4.4. Smart Functions, Repair, and Circular Pallet Systems

Digital manufacturing makes it straightforward to incorporate cavities, channels, labels, mounting points, and protective housings for tracking or sensing hardware. Related work on intelligent packaging, embedded functionality, and AM for electronics demonstrates the broader feasibility of integrating digital features into printed structures [30,31,107]. In a pallet context, this could support RFID protection, shock logging, temperature sensing, or traceability without adding separate external brackets.

**Figure 6:**
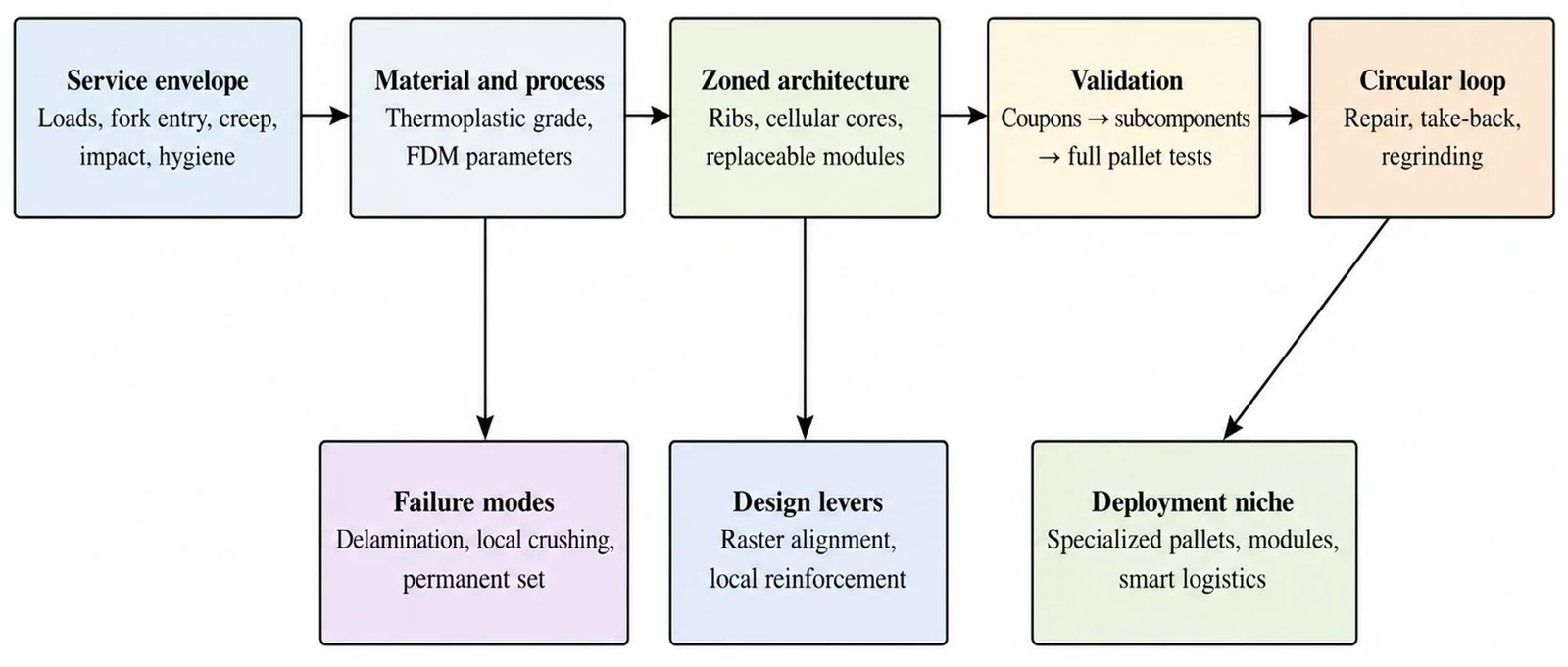
Pallet-oriented design and validation pathway linking service definition, AM material-process selection, zoned architecture, qualification, and circular recovery.

The sustainability value of “smart” features should be judged by the decisions they enable. A sensor that detects overloading, impact, moisture exposure, or remaining service condition may extend pallet life by preventing premature disposal or unsafe reuse. Conversely, embedding electronics in a way that prevents separation at the end of life can complicate recycling. Design for disassembly is therefore important: electronics should ideally be removable while the polymer body remains recoverable. Figure 6 consolidates the pallet-development pathway from service requirements to zoned architecture, process-aware design, structural qualification, and circular recovery [12,34,66,68,69,70,71,72,73,74,75,76,77,80,84,95].

Repair is often environmentally preferable to full material recycling because it preserves the embodied energy and shape of the existing product. AM can support repair through printed replacement modules, polymer welding, local deposition, or interchangeable sacrificial components. Pallets are particularly amenable to this strategy because edge, foot, or fork-entry damage may be localized. A modular design can replace the damaged region while retaining the rest of the structure.

Design for disassembly should also govern smart components. Sensors, tags, or metal fasteners should be removable before polymer recycling. Multi-material architectures should be used only when their functional benefit outweighs the difficulty of separation. These principles are consistent with sustainable design guidelines for AM and with the wider shift from linear to circular manufacturing [30,108].

The evidence therefore supports a clear distinction between demonstrated AM process capability and pallet-specific performance. Graded cores, sacrificial fork-entry regions, modular joints, repairable zones, and integrated sensing are technically consistent with broader polymer-AM evidence, but they remain to be validated at pallet scale under rack loading, repeated handling, impact, creep, environmental conditioning, sanitation requirements, and logistics-system compatibility. Table 2 consolidates these requirements as an evidence-informed translation framework rather than as a record of already qualified AM pallets.

### 4.5. Scale-Up and Pallet-Specific Evidence Gaps

Pallet research lacks a sufficiently mature bridge between coupon optimization and full structural qualification. Many FDM studies focus on tensile or flexural coupons, yet pallets are governed by structural load paths, local contact, impact, creep, and repeated handling. Future work should develop component-specific material models that incorporate print orientation, infill, local bead architecture, temperature, and time-dependent behaviour [66,80,81,82,84,89].

Full-scale or representative subcomponent tests should include deck bending, runner or foot compression, fork-entry impact, repeated handling, creep under sustained load, and environmental conditioning. Failure analysis should be used to refine geometry rather than simply report the ultimate load. Finite-element simulation should be calibrated using process-specific properties and validated at the component level. The goal is to establish design allowables and safety factors that are meaningful for AM structures.

Large-format print economics and energy use are another gap. Build time, machine utilization, material cost, labour, failure risk, and repair must be compared with conventional pallet supply chains. Recycled thermoplastic should be evaluated over multiple reprocessing cycles to quantify property drift and to define controlled blending strategies. A genuinely circular AM pallet requires not only recyclability in principle but a validated collection, sorting, reprocessing, and quality-assurance loop [7,9,44,45].

Vat-photopolymerization pallets remain primarily a conceptual or prototype-level opportunity due to limited build volume, high resin costs, brittleness, and recycling limitations. Future work should focus on the specific pallet functions for which high resolution or resin chemistry creates unique value, rather than forcing whole-pallet fabrication. Examples include modular joints, protective inserts, sensor housings, high-detail identification elements, or specialized cleanroom and low-volume transport solutions.

When structural resin parts are considered, mechanical assessment should extend beyond short-term strength to account for impact, fatigue, creep, UV exposure, humidity, and temperature. Resin toughness and post-cure conditions must be reported in enough detail to reproduce performance. Sustainable resin development should simultaneously address renewable content, lower toxicity, repairability, and end-of-life treatment [6,22,23,40,58,65,91].

Build size is a central scale-up constraint. Desktop FDM coupons can demonstrate a lattice or material concept but do not establish the thermal behaviour, productivity, distortion, or structural repeatability of a pallet-scale build. Large-format systems change bead size, cooling history, toolpath length, void structure, and dimensional accuracy; design allowables should therefore be generated for the actual manufacturing scale rather than transferred from small-nozzle coupons [68,69,78].

Large-format FDM or pellet extrusion is consequently the most relevant manufacturing evidence for pallet-scale translation. Its higher deposition rate can reduce build time, but coarse beads alter achievable cellular geometry, inter-bead contact, surface finish, and local stress concentration. Pallet-scale architectures should therefore be designed around large-format process physics from the start. Until full-scale structural testing and lifecycle economics are reported, claims of lower mass, lower impact, or superior durability should remain prospective rather than definitive [68,69,77,78,89].

## 5. Additive Manufacturing for Membrane Feed Spacers

### 5.1. Functional Role and Geometry–Performance Coupling

Feed spacers maintain the channel between membrane sheets in spiral-wound modules and strongly influence flow distribution, pressure drop, concentration polarization, mass transfer, and fouling. Conventional spacers are often mesh-like structures designed primarily for manufacturability and mechanical separation. Their geometry can create wakes, recirculation zones, local high-shear regions, and low-velocity dead zones. Because the spacer occupies part of the flow channel, any improvement in mixing must be balanced against additional hydraulic resistance [16,109,110,111,112,113,114,115,116,117,118].

This trade-off makes feed spacers particularly well suited to AM-enabled design exploration. Conventional extrusion and bonding constrain filament shape and crossing geometry, whereas AM can vary filament diameter, angle, curvature, cross-section, pitch, surface texture, and three-dimensional connectivity. The designer can therefore search for geometries that enhance near-wall mixing without producing an excessive pressure-drop penalty. This is a fundamentally different sustainability mechanism from pallet lightweighting: the greatest environmental benefit may occur during use through reduced demand for pumping or cleaning rather than through reduced mass alone [13,16,17].

Critical spacer variables include channel height, filament diameter, filament orientation, crossing angle, pitch, blockage ratio, surface roughness, and the spatial arrangement of contact points. Curved, sinusoidal, helical, or biomimetic features can deliberately generate secondary flow and redistribute shear. TPMS-inspired structures constitute another design family, as smooth periodic surfaces can produce continuous three-dimensional pathways without the sharp filament crossings found in conventional meshes [48].

The relationship between geometry and performance is not monotonic. Increasing obstruction can increase mixing and mass transfer but also increase pressure drop. Increasing surface roughness can disrupt boundary layers but may also create nucleation sites for fouling. Narrower channels can increase velocity at a given flow rate but increase pumping demand. Therefore, spacer optimization should be multi-objective, with at least pressure drop, mass-transfer enhancement, fouling tendency, manufacturability, and cleanability considered simultaneously.

AM enables the creation of graded spacers in which the geometry changes along the flow direction. For example, stronger mixing could be introduced near regions prone to concentration polarization while lower-resistance sections are used elsewhere. Functionally graded manufacturing has been demonstrated in material-extrusion contexts [86], and the same design philosophy can be extended to spatially graded flow structures. The key challenge is to prove that the added geometric complexity yields a net operational benefit sufficient to justify the added manufacturing complexity.

### 5.2. Process Selection and Material Compatibility

Vat photopolymerization is attractive for feed spacers because it can produce smaller, smoother features than those produced by typical filament extrusion. DLP is particularly efficient for repeated lattice-like structures, while SLA offers fine scanning control. DLP-based microfluidic fabrication can produce complex internal channels and small features that are difficult to manufacture conventionally [119]. Reviews of 3D printing in water treatment similarly identify high-resolution photopolymerization as an important route for customized membranes, supports, and spacer structures.

The main limitation is material suitability. A spacer remains in prolonged contact with water and may experience oxidants, acids, bases, surfactants, or cleaning agents. Cured resin must therefore retain dimensions and mechanical integrity while avoiding unacceptable leaching. Standard photopolymer resins developed for prototyping cannot automatically be assumed suitable for water-treatment service. Resin selection, complete cure, solvent extraction, ageing, and chemical-resistance testing are central to translation [120,121,122,123].

Ceramic DLP provides an alternative where chemical stability and rigidity are important [24,63,94]. Ceramic structures can withstand environments that would degrade some polymers, but debinding and sintering introduce brittleness risk, shrinkage, and substantial thermal-processing energy. Their sustainability case is therefore application-specific and should be evaluated against service-life extension rather than geometric capability alone.

FDM offers lower equipment cost and easier thermoplastic material handling, making it attractive for rapid spacer prototyping and larger channel features. Water-treatment literature has explored 3D-printed membranes, supports, and related flow structures using polymer AM [16,17,124]. The principal limitation is resolution. Nozzle diameter and bead width constrain minimum filament dimensions, while stair-stepping and inter-road gaps can alter local roughness and channel shape.

Surface roughness can be either beneficial or detrimental. Moderate roughness may increase mixing, but irregular deposition can produce uncontrolled dead zones and trap foulants. For this reason, FDM spacer studies should distinguish intentionally designed roughness from process-induced defects. Dimensional metrology and surface characterization are essential before interpreting hydraulic performance. A nominal CAD geometry alone is insufficient because the actual printed channel controls fluid behaviour.

Thermoplastic FDM has a circularity advantage because spacers could potentially be mechanically recycled. Yet the chemical purity requirements of membrane service may restrict the use of mixed or contaminated recycled feedstock. Material selection must also account for swelling, hydrolysis, temperature, cleaning chemicals, and biofilm interactions. The most sustainable spacer is not necessarily the one with the highest recycled content; it is the one that maintains function, limits leaching, and achieves a favourable life-cycle balance.

For membrane feed spacers, prolonged water contact creates additional material requirements. Residual monomer, photoinitiator fragments, oligomers, and additives may leach if cure is incomplete or the network degrades. Degree of conversion, extraction behaviour, post-cure protocol, swelling, and long-term immersion stability therefore become qualification variables. A resin that prints with excellent resolution may still be unsuitable if it embrittles, swells, or releases extractables. Table 3 summarizes the principal geometric/material design variables and their competing hydrodynamic or durability effects [6,13,14,15,16].

### 5.3. Hydraulics, Fouling, Cleaning, and Performance Validation

Fouling is dynamic, and short hydraulic tests cannot fully predict long-term spacer performance. Biofouling, particulate deposition, scaling, and organic fouling interact differently with surface chemistry and local flow. A geometry that produces high initial mass transfer may later develop severe deposits at stagnation points. Long-duration experiments are therefore necessary to connect early pressure-drop measurements with fouling evolution and cleaning recovery [125,126,127,128,129,130,131].

Cleaning adds another design dimension. High-shear and well-mixed channels may reduce deposit formation, but complex internal cavities can be difficult to clean once fouled. Spacer geometry should therefore be evaluated for both fouling resistance and cleanability. Repeated chemical-cleaning cycles should also be used to assess material swelling, embrittlement, surface change, and leachability. These lifecycle tests are especially important for photopolymer-based spacers [13,14,15,16].

Performance should be reported with normalized quantities that permit comparison across geometries and operating conditions: pressure-drop gradient, friction factor, mass-transfer enhancement, specific water flux, flux decline, fouling-layer thickness or biomass accumulation, cleaning recovery, and energy-normalized water productivity. The published primary literature already demonstrates that these quantities can distinguish useful geometry from visual complexity; the main weakness is inconsistent reporting of channel dimensions, materials, and baselines across studies [115,125,128,132,133,134,135].

Feed-spacer evaluation should similarly progress from geometric characterization to clean-water hydraulics, mass transfer, fouling, and cleaning recovery. Initial hydraulic tests should establish pressure drop over a defined channel length and operating range. The data should be normalized where possible using channel geometry and flow conditions so that different spacer designs can be compared fairly. A lower pressure drop is beneficial only if mass transfer and fouling performance remain adequate.

Flow visualization or computational fluid dynamics can help explain why a geometry performs as observed. Velocity fields, recirculation zones, wall shear, residence-time distribution, and local turbulence or secondary flow are useful descriptors. However, simulations should use measured rather than idealized dimensions when manufacturing deviations are significant. Model assumptions should also be validated against pressure-drop or tracer data rather than treated as self-evident.

Fouling tests should distinguish among organic fouling, particulate deposition, scaling, and biofouling because the controlling mechanisms differ. A spacer that performs well against one foulant may not generalize to another. Long-duration experiments should track flux decline, transmembrane pressure, pressure drop, deposit distribution, and cleaning recovery. The geometry should be inspected after testing to determine where deposits accumulate relative to predicted low-shear zones.

**Figure 7:**
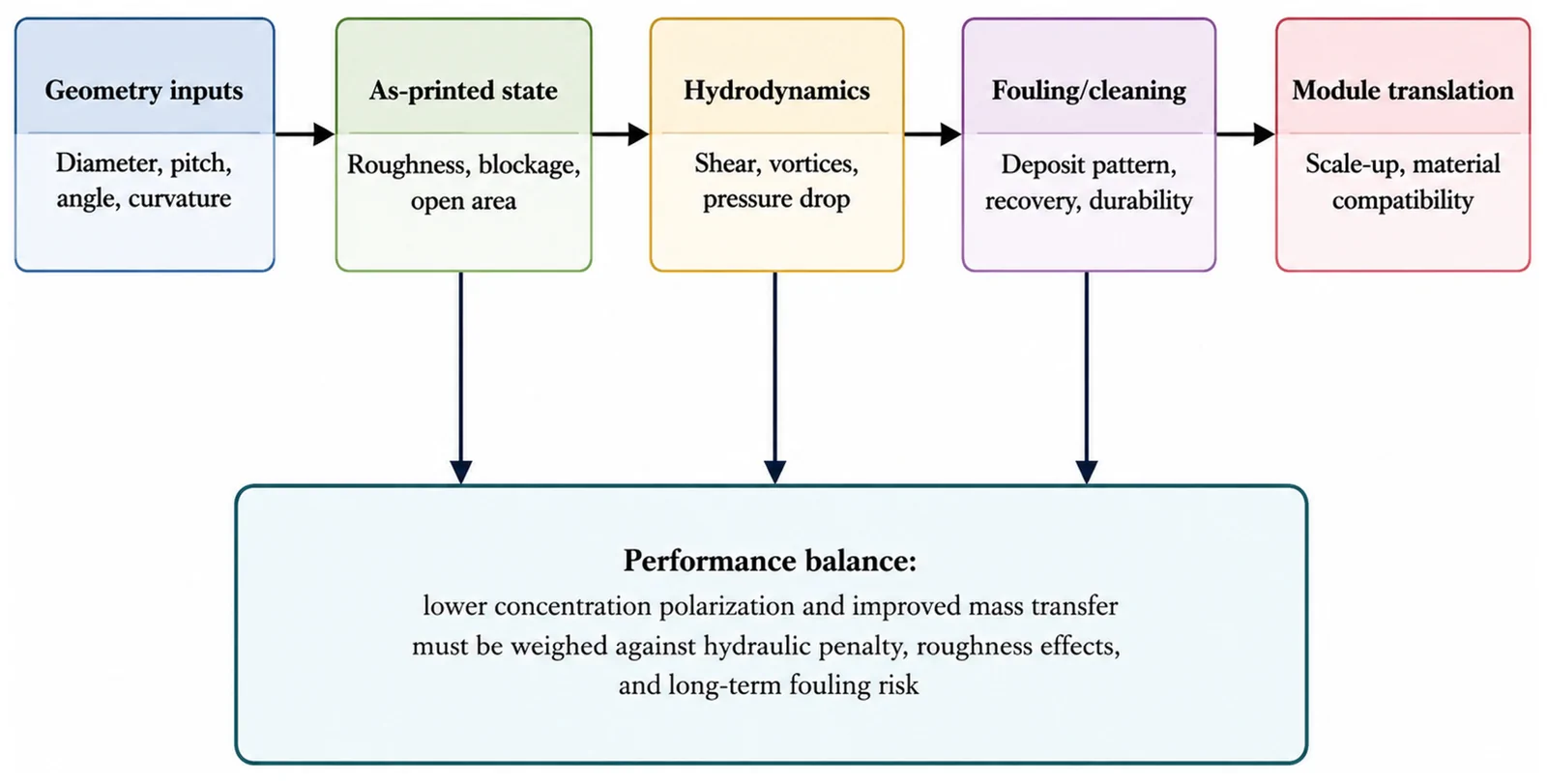
Feed-spacer geometry–performance pathway linking design variables to hydrodynamics, fouling behaviour, cleaning response, and module-scale translation.

Chemical cleaning is part of the operating life and should be treated as a durability test. Repeated exposure can alter polymer dimensions, surface chemistry, brittleness, or interfacial bonding, particularly in photopolymer spacers. The strongest current evidence nevertheless shows that geometry can materially alter hydraulic and fouling behaviour: perforated designs have reported 50–61% lower pressure drop than a non-perforated printed baseline [128]; a 3D-printed column spacer reduced pressure drop by about threefold and doubled specific water flux [133]; a hole-pillar design reported a pressure-drop gradient of 0.091 ± 0.009 bar m^−1^ at 0.185 m s^−1^ compared with 0.118 ± 0.009 bar m^−1^ for a commercial spacer [132]; and a honeycomb spacer produced a 119.0 μm fouling layer versus 175.5 μm for a standard diamond geometry while increasing permeate flux by 16% [134]. Table 4 consolidates the quantitative evidence and also identifies where dimensions or material details are not consistently reported. Figure 7 summarizes the geometry-to-performance pathway [13,14,15,16,17,109,110,111,112,113,114,115,116,117,118,125,126,127,128,129,130,131,132,133,134,135,136].

### 5.4. Translation from Laboratory Coupons to Membrane Modules

Feed-spacer research therefore contains substantially more direct application evidence than the pallet domain, but the studies remain difficult to compare because printer type, material, channel height, filament size, flow regime, membrane area, water chemistry, and baseline spacer differ. Table 4 shows both the strength and the limitation of the evidence: several studies report meaningful pressure-drop, flux, or fouling improvements, yet the reported geometry and operating conditions are not standardized enough for a universal ranking of spacer topology.

Future studies should therefore integrate CAD geometry, as-printed metrology, CFD or analytical prediction, pressure-drop measurement, mass-transfer performance, fouling development, and cleaning recovery into a single experimental framework. Repeated-use tests are needed to establish whether performance persists after chemical cleaning and ageing. For photopolymers, leachability and conversion should be treated as mandatory qualification parameters rather than optional material details.

Another gap is the translation of small laboratory coupons into spiral-wound modules. Scale-up can change flow distribution, boundary conditions, deformation, and manufacturing defects. Research should progressively move from idealized cells to larger membrane areas and module-relevant geometries while retaining adequate diagnostic access. DLP and SLA are well suited to rapid geometry screening; FDM may provide a path to larger prototypes. Hybrid strategies could therefore use high-resolution printing for design discovery and more scalable methods for final manufacturing [132,133,134,135,136].

Taken together, the feed-spacer literature supports geometry as an active process variable rather than a passive separator shape. Confidence is strongest for laboratory-scale hydraulic and short-to-intermediate fouling trends and weaker for long-term chemical ageing, repeated cleaning, leachability, and module-scale translation. Future claims should therefore report as-printed dimensions, pressure-drop gradient, mass-transfer or flux response, fouling evolution, cleaning recovery, material ageing, and leachability together rather than using a single favourable metric [115,125,128,132,133,134,135,136].

## 6. Cross-Scale Critical Synthesis, Evidence Quality, and Validation

### 6.1. Material Efficiency, Use-Phase Performance, and Circularity Across Scales

Both pallets and feed spacers can use less material through AM, but the significance of material reduction differs. For pallets, component mass is large enough that lightweighting can directly influence material demand and, potentially, transport energy. For spacers, absolute material savings are comparatively small, so the dominant lifecycle benefit may come from operational efficiency. This difference demonstrates why sustainability metrics must be selected according to functional unit rather than applied uniformly [1,5,10].

Material efficiency also depends on supports, failed builds, purge waste, machining allowance, washing, curing, and rejection. A low-density lattice that requires extensive support can lose much of its theoretical material advantage. Likewise, an intricate resin geometry that frequently fails or requires solvent-intensive cleaning can be less sustainable than a simpler geometry with a high manufacturing yield. Robustness and process capability are therefore sustainability variables, not only quality-control variables [1,5,8,10].

The most important cross-application insight is that AM should be justified by service performance. In pallets, this may be mass reduction, durability, repairability, or digital functionality. In membrane spacers, it may be lower pressure drop at comparable mass transfer, delayed fouling, longer membrane life, or reduced cleaning frequency. These use-phase benefits can outweigh manufacturing burdens when they are sufficiently large and persist over the product lifetime [4,5].

This principle also explains why direct “AM versus conventional process” comparisons can be misleading when the geometry is held constant. If AM simply reproduces an injection-moulded spacer or conventional pallet geometry, its primary advantages are underused. The appropriate comparison is between optimized product systems that perform the same function. Design for additive manufacturing is therefore central to sustainability assessment [34].

FDM has a relatively direct circularity pathway because thermoplastics can be mechanically reprocessed, although degradation and contamination must be controlled [7,45]. SLA/DLP systems can reuse uncured resin to some extent, but cured cross-linked polymers are more difficult to remelt or mechanically recycle. This does not make vat photopolymerization unsustainable, but it shifts the strategy toward resin efficiency, long service life, low-toxicity chemistry, bio-derived feedstocks, and emerging chemical-recycling concepts [6,40,65].

The end-of-life strategy should influence design from the beginning. Mono-material structures are easier to recover than inseparable multi-material assemblies. Replaceable modules may extend the service life of a pallet. Removable electronics can preserve polymer recyclability. For spacers, longer life and lower cleaning demand may reduce replacement burden even if direct material recycling is limited. Circular design is therefore broader than recyclability alone.

AM is compatible with digital supply chains, decentralized production, rapid design iteration, and traceable process data [11,35]. Pallets could benefit from local manufacturing of specialized or replacement parts, while membrane research can use digital libraries of spacer geometries that are produced consistently across laboratories or pilot facilities. Digital manufacturing also enables design updates without new tooling.

Yet distributed manufacture can only support sustainability if quality is repeatable. Digital design files should therefore be accompanied by validated material grades, process parameters, calibration requirements, inspection methods, and acceptance criteria. Otherwise, decentralized production may increase variability and rejection rates. Integration of process monitoring, data analytics, and closed-loop control is an important future direction to ensure that digitally distributed components maintain qualified performance.

### 6.2. As-Printed Characterization, Reproducibility, and Uncertainty

AM components should be characterized as manufactured, not only as designed. The deposited or cured geometry can deviate from CAD because of bead width, shrinkage, cure overgrowth, support removal, thermal distortion, surface roughness, stair-stepping, and local defects. In a pallet, these deviations may alter section modulus, contact geometry, and crack initiation. In a spacer, they may alter open flow area and therefore pressure drop. The as-printed state is therefore the true input to performance interpretation.

A minimum dimensional characterization plan should include external dimensions, critical feature sizes, flatness or warpage, thickness variation, and local surface condition. For lattice or spacer architectures, imaging should quantify filament diameter, node geometry, pore/channel dimensions, and any blocked or partially formed regions. When internal geometry is inaccessible, non-destructive methods or representative sectioning may be required. The purpose is not simply quality control; it is to establish whether performance trends arise from the intended design or from manufacturing deviation.

Surface roughness deserves separate attention because it plays different roles in the two applications. In pallets, roughness can influence friction, handling, cleanability, and wear but may be secondary to bulk structure. In membrane spacers, roughness can directly affect boundary-layer development, particle attachment, and biofilm initiation. Therefore, reporting a single average roughness value without linking it to flow orientation or surface morphology may be insufficient. Surface characterization should be selected according to the physical mechanism being investigated.

Reviewed AM research is often difficult to compare because machine settings, material condition, build orientation, post-processing, and specimen geometry are reported inconsistently. Reproducibility requires more than naming the printer and material. At minimum, layer thickness, nozzle or pixel scale, temperature or exposure settings, build orientation, support strategy, post-cure or annealing conditions, and conditioning before testing should be provided where relevant.

Multiple builds should be used when feasible because within-build repeatability does not capture day-to-day process variation. Statistical reporting should include sample size and variability rather than only mean values. For optimization studies, confirmation experiments are important because the optimum predicted by a statistical or machine-learning model may be sensitive to noise or untested interactions. These practices improve scientific confidence and also reduce sustainability risk by revealing process windows that are robust rather than narrowly optimized.

Uncertainty should be propagated into component design. If recycled filament exhibits greater property variation than virgin material, structural safety factors or grading criteria may need adjustment; if printed spacer dimensions vary, the resulting spread in pressure drop should be quantified. This approach transforms manufacturing variability into a design parameter that can be managed. Table 5 consolidates characterization, qualification, and translation requirements for both application domains.

### 6.3. What the Combined Literature Establishes

The combined literature establishes three evidence levels rather than one uniform body of proof. First, process-level evidence is strong: polymer AM properties and energy use depend on layer/exposure settings, thermal history, orientation, deposition rate, build utilization, and post-processing [1,2,3,4,5,6,7,8,9,10,18,25,51,52,53,62,70,71,72,73,74,75,76,77,78,79,80,81,82,83]. Second, direct feed-spacer evidence is moderate to strong at laboratory scale because printed geometries have been experimentally linked to pressure drop, flux, and fouling [115,128,132,133,134]. Third, direct whole-pallet AM evidence is weak because the reviewed literature does not contain a fully qualified printed transportation pallet. Pallet conclusions must therefore be treated as evidence-informed translation requirements, not demonstrated product superiority.

This asymmetry changes how sustainability should be interpreted. A pallet-scale design is a relatively large structural system whose lifecycle burden can be influenced by material mass, build energy, transport frequency, damage rate, repair, and recovery. A feed spacer has little mass but can affect pumping energy, cleaning chemicals, membrane replacement, and water productivity. Applying one indicator to both would therefore obscure the service function. Functional units must reflect payload/service cycles for pallets and treated-water or membrane-productivity service for spacers [5,10].

The evidence also shows that geometric freedom only creates environmental value when it changes service performance. If AM merely reproduces a conventional pallet or mesh without lowering mass, avoiding tooling, improving durability, reducing pressure drop, or enabling repair, its higher machine or post-processing burden can dominate. Conversely, a more energy-intensive print can be justified when the resulting architecture produces a durable and measurable service benefit. This is the central critical distinction between design capability and sustainability outcome [1,2,5,34].

The literature also highlights a recurring methodological limitation: many comparisons are sensitive to system boundaries and assumptions. Energy per kilogram, energy per part, and environmental impact per functional service can yield different rankings. Machine utilization and build-to-order packing can change the outcome, as can the electricity mix and the assumed lifetime of the printed product. Accordingly, future pallet and spacer assessments should report their functional unit, system boundary, material yield, build utilization, and lifetime assumptions explicitly [1,2,5].

The SLA/DLP literature shows a clear evolution from geometric prototyping toward engineered materials. SLA process studies demonstrate robust, high-resolution fabrication, while orientation-dependent mechanical work shows that the performance of printed resin remains sensitive to build direction and ageing [19,20,21]. Nanoparticle-reinforced systems then extend the mechanical and thermal envelope, with metal–metal-oxide and silica-containing composites illustrating how fillers can strengthen the cured network when dispersion and cure remain controlled [22,58]. Thiol–acrylate chemistry and stretchable DLP elastomers show that network chemistry can also be adjusted to increase flexibility and reduce the brittleness associated with conventional photopolymers [23,90].

Across these vat-photopolymerization studies, one consistent gap is the lack of application-specific long-term durability. High resolution and improved initial properties are well established, but fewer studies connect resin chemistry to repeated chemical exposure, leaching, long-term water immersion, fatigue, or repair. This gap is central to feed-spacer deployment and to any structural resin component used in logistics. The next generation of studies should therefore move from printability and short-term property enhancement toward lifetime qualification.

The FDM literature is more mature in process-parameter and mechanical-property relationships. Reviews and experimental studies repeatedly identify layer thickness, infill, raster strategy, temperature, speed, and orientation as dominant variables that shape mechanical response [73,78,79,80,81,82,83]. This body of evidence is directly useful for pallet design because it demonstrates that mechanical properties can be tuned, but also that they cannot be separated from manufacturing settings. It also explains why bulk polymer data are insufficient for structural simulation.

Anisotropy emerges as a persistent issue. Material-extrusion structures behave differently along and across deposition directions, and composite FDM can amplify directional behaviour depending on reinforcement alignment [84]. This does not merely represent a defect; it can be used as a design variable. If toolpaths are aligned with major load paths, material can be placed efficiently. The research opportunity is therefore to move from avoiding anisotropy to managing it deliberately through process-aware structural design.

Composite FDM literature shows substantial potential to improve stiffness, thermal stability, wear, and functional performance [66,67]. Yet the studies also imply trade-offs in flow, interlayer bonding, nozzle wear, and recyclability. For pallet applications, composite material should be deployed where it reduces mass or increases life sufficiently to offset these burdens. For spacer applications, fillers can alter surface properties or adsorption but can also increase roughness, leaching risk, or contamination. A consistent sustainability assessment should therefore evaluate the complete composite system, not only its improved mechanical properties.

Recycled-filament literature provides one of the clearest pathways toward circular AM. Reviews of waste-plastic filament and recycled thermoplastics describe a technically feasible route from waste collection through shredding, extrusion, and reprinting [44,45]. Comparative sustainability studies show that recycled feedstock can reduce certain material-related impacts but must be evaluated for energy use and health considerations [7]. Recent studies on recycling and polymer sustainability reinforce the need to quantify property retention rather than assume infinite recyclability [9].

The circularity literature also highlights a distinction between open-loop and closed-loop recycling. Open-loop use of heterogeneous waste can provide environmental value, but often produces variable feedstock. Closed-loop systems, in which a known material stream is collected and reprocessed, offer greater control and are better suited to structural pallets. This suggests a specific research model: use logistics networks not only to move pallets but also to recover and requalify their material at the end of life. Such a system could convert the distributed nature of pallets from a recycling challenge into a circular-economy opportunity.

The water-treatment literature establishes that 3D printing is more than a fabrication convenience: it can control pore architecture, channel connectivity, flow distribution, surface exposure, and component integration [13,14,15,16,17,115,119,135]. Its most direct sustainability value appears when those geometric changes reduce hydraulic resistance, fouling, or cleaning burden without sacrificing mass transfer or material compatibility.

For feed spacers, filament angle, diameter, pitch, curvature, surface texture, and three-dimensional connectivity influence wall shear, pressure loss, residence time, and dead-zone formation. AM expands this design space, but the quantitative studies in Table 4 show that improvements are geometry- and condition-specific rather than universal [115,125,128,132,133,134].

DLP and SLA are particularly aligned with this need because fine features and smooth surfaces can be fabricated with high fidelity. Microfluidic DLP demonstrates the broader capability to manufacture small channels without multi-step assembly [119], while ceramic DLP membrane work indicates that complex porous structures can be translated into chemically stable materials [63]. FDM offers a lower-cost route and is valuable for rapid prototyping, but nozzle size and bead morphology limit the minimum feature size.

Water-treatment reviews also identify a translation challenge: many AM materials are developed for prototyping rather than prolonged fluid contact [13,14,16]. The literature highlights chemical compatibility, swelling, mechanical stability, surface interactions, and potential leaching. These issues are especially important because a material acceptable for a dry mechanical part may be inappropriate for water purification. The evidence, therefore, supports a two-stage development pathway in which geometry is optimized first and then reproduced in a qualified material system.

Overall evidence quality is therefore application-dependent. The pallet-scale side has high confidence in enabling principles such as anisotropic FDM behaviour, large-format extrusion, lattice mechanics, and thermoplastic recycling, but low confidence in whole-pallet AM performance until component-scale validation is available. The feed-spacer side has stronger direct laboratory evidence for hydrodynamic and fouling effects, but lower confidence in long-term leaching, cleaning-cycle durability, and full-module scale-up. This evidence grading is used in the conclusions so that no application claim exceeds the support available in the cited literature [66,67,68,69,70,71,72,73,74,75,76,77,78,79,80,81,82,83,84,115,125,128,132,133,134,135,136].

## 7. Life-Cycle Assessment, Circular Economy, and Techno-Economic Translation

### 7.1. Functional Units and Life-Cycle System Boundaries

A life-cycle assessment of AM should include more than raw feedstock and printer electricity. The system boundary can include feedstock production, filament extrusion or resin formulation, packaging, transport, printer warm-up and standby, build energy, support material, failed builds, post-processing, machining, solvent washing, post-curing, assembly, distribution, service energy, maintenance, cleaning, repair, replacement, and end-of-life treatment. The relative importance of these stages changes dramatically between a pallet and a feed spacer [137,138].

For pallets, raw material, printing energy, build time, transportation, service life, repair, and end-of-life recovery are likely to dominate. The use-phase mass benefit depends on how the pallet is transported relative to the payload and over how many cycles. For spacers, component mass is small, so the operational energy of pumping and the frequency of cleaning or membrane replacement may dominate. A sustainable spacer can therefore justify a more intensive manufacturing route if it reliably reduces system-level energy or maintenance.

This difference reinforces the importance of a functional unit. Comparing impacts per kilogram of printed material can favour one technology while obscuring actual service value. A pallet should be assessed according to the logistics function it serves, whereas a spacer should be assessed according to the membrane-treatment service it provides. Functional units encourage designs that achieve the required function with the lowest total resource burden rather than merely the lowest manufacturing mass.

### 7.2. Energy Accounting and Machine Utilization

AM energy intensity is strongly affected by machine utilization, build packing, deposition or exposure rate, warm-up, standby, failed builds, and post-processing. Because the literature uses different functional units, the reported values should not be collapsed into a single process ranking. Hopkins et al. reported desktop volumetric specific energy consumption of 24.8–85.7 kJ cm^−3^ for FFF, 10.8–21.5 kJ cm^−3^ for SLA, and 18.4–19.3 kJ cm^−3^ for an area-exposure vat-photopolymerization system under the tested conditions [51]. Lunetto et al. reported 9.47 MJ (2.63 kWh) for a 149.5 g FDM validation build and a modelled specific energy of 66.2 MJ kg^−1^ (18.4 kWh kg^−1^) under their study conditions [52]. Real production can be materially worse than idealized estimates: Song and Telenko measured 0.835 MJ kg^−1^ for preheating, 21.5 MJ kg^−1^ for printing, and 9.5 MJ kg^−1^ for standby in a commercial FDM setting, while their life-cycle inventory was about 50% higher than the ideal case because of operating losses and failed builds [139]. Faludi et al. further showed that machine utilization and idle-energy allocation can change the lifecycle comparison; in their tested high- and low-utilization scenarios, the FDM machine had the lowest ecological impact per part among the FDM, PolyJet, and CNC systems compared [137]. Table 6 preserves the functional unit and boundary reported by each study instead of creating an artificial cross-process ranking [8,51,52,53,64,137,139,140].

Energy optimization should target useful function per unit resource rather than the lowest instantaneous printer power. For pallet-scale FDM, larger nozzles and higher deposition rates can reduce machine time but also coarsen geometry and alter bonding. For SLA/DLP, increasing layer thickness or exposure efficiency can reduce build time but may change dimensional accuracy and cure. The process optimum is therefore constrained by minimum acceptable structural or hydrodynamic performance, dimensional fidelity, and build yield [51,52,53,64].

For SLA/DLP, washing and post-curing must be included explicitly because the printing stage is only one part of the inventory. Solvent recovery, resin filtration, bath replacement, cure energy, rejected resin, and cured thermoset end-of-life can materially change the result. Recent lignin-resin LCA work also demonstrates the importance of upstream material production: a 10 wt% lignin-blended resin was reported at 6.64 kg CO_2_-eq kg^−1^ resin, with the commercial epoxy fraction remaining a dominant contributor [60]. These values should be read as study-specific evidence with defined boundaries, not universal process constants.

### 7.3. Circular Material Loops, Repair, and Design for Disassembly

Circularity is strongest when material identity and quality are preserved, and recycled content should therefore be evaluated together with both process emissions and retained performance. Yang et al. showed that orientation angle, layer height, and heated-bed temperature were dominant variables for recycled-filament printing; multi-objective parameter optimization improved the combined objectives by 20.24%, reduced particulate-matter impact by 54.02%, and lowered lifecycle emissions by 21.7% under the reported conditions [7]. Other studies show that mechanical retention can range from limited loss to substantial degradation depending on polymer type, waste source, thermal history, blending, drying, and printing conditions [9,87,88,141,142,143,144]. For structural pallet-scale concepts, this variability must be translated into feedstock acceptance criteria, process windows, and design allowables rather than absorbed into an unspecified safety factor.

Closed-loop logistics may provide a practical advantage because pallets are already tracked, collected, and redistributed in many systems. If polymer grade and recycling history are encoded and the structure is designed for disassembly, damaged modules could be separated and reprocessed. The viability of this model depends on whether collection and reprocessing energy, labour, contamination control, and property recovery are justified by the recovered material value and the service-life extension.

For cured photopolymers, circularity is more difficult because cross-linked networks do not simply remelt. Strategies therefore shift toward resin minimization, recovery of uncured material, longer service life, renewable constituents, lower-toxicity chemistry, and emerging chemically recyclable networks [6,40,59,60,65]. Table 7 summarizes representative quantitative recycled-polymer trade-offs and shows that environmental benefit and mechanical retention must be reported together.

For feed spacers, repair is less likely to be practical because components are thin and integrated into membrane modules. Circularity may instead be achieved through longer service life, lower replacement frequency, simplified material composition, and recoverable thermoplastic designs where water-quality requirements permit. Again, the relevant circular strategy depends on the component rather than on a generic AM principle.

### 7.4. Industrial Production, Qualification, and Techno-Economics

The transition from laboratory feasibility to industrial production requires stable yield. A process that produces excellent parts intermittently can be environmentally and economically poor because failed builds consume material, machine time, post-processing, and labour. Production readiness therefore requires a validated process window, not one optimized parameter set. Material conditioning, calibration, environmental control, preventive maintenance, operator procedures, and acceptance inspection should be treated as part of the manufacturing system.

Industrial users require predictable acceptance criteria. For pallets, qualification should define allowable deflection, failure load, impact tolerance, repeated-use performance, environmental conditioning, and dimensional compatibility with handling systems. For feed spacers, qualification should define geometry, pressure-drop performance, chemical stability, leachability, fouling response, and cleaning durability. The exact test standard may differ by application, but the principle is the same: a printed part should be accepted on measured functional performance rather than on printer settings alone.

Process qualification should link material lot, machine, software, parameters, build orientation, and post-processing to final properties. This is particularly important for distributed production because nominally identical files may be manufactured on different machines. A digital build record can provide traceability and can support root-cause analysis when a component fails. Industry 4.0 integration makes this digital thread increasingly feasible [35].

Recycled material adds another layer of qualification. The recycled fraction, source, number of reprocessing cycles, moisture, contamination, and rheology should be controlled. A circular material loop without specification can create inconsistent performance and excessive rejection. Qualification can therefore increase sustainability by making recycled feedstock sufficiently dependable for repeated use, rather than restricting it to low-value applications.

The economic case for AM depends on more than material price. Machine depreciation, energy, labour, build preparation, post-processing, quality inspection, failure rate, maintenance, and throughput all contribute to part cost. AM avoids dedicated tooling, which favours low-volume, customized, or rapidly changing designs, but conventional moulding can remain far more economical at high volume. The most credible deployment strategy is therefore likely to be application selective.

For pallets, high-volume commodity production creates a demanding cost target, so AM may first be viable for specialized structures, replacement modules, low-volume logistics, tooling, or smart features rather than direct mass-production substitution. For feed spacers, the value proposition can be stronger in research, pilot-scale, speciality separations, or high-value treatment systems where geometry-driven pressure-drop, flux, or fouling improvements can offset higher component cost [132,133,134,135]. Economic comparison should therefore include operational savings over service life rather than unit part price alone.

Techno-economic models should therefore be coupled with lifecycle models. Lower environmental impact at an unaffordable cost may not be adopted, while a cheaper printed component with poor durability can increase both cost and waste. Joint environmental-economic optimization can identify designs that are realistically deployable and can expose which technical improvements, such as faster deposition, longer service life, or lower resin cost, would have the greatest impact on adoption.

Hybrid manufacturing offers a practical bridge between AM flexibility and conventional productivity. A pallet may combine extruded or moulded primary members with AM-optimized connectors, reinforced corners, or damping elements. A membrane module may combine conventionally manufactured membranes with AM spacers or distributors. This approach avoids using AM for geometrically simple mass while preserving its value for complex features.

Staged adoption is also useful for risk management. AM can first be used for rapid prototyping and design screening, then for pilot components, then for low-volume service, and finally for larger production if durability and economics are demonstrated. Such a pathway is more realistic than assuming immediate replacement of mature manufacturing processes. It also generates the data needed for standards, process qualification, and life-cycle assessment. Ultimately, AM should be treated as one element of a manufacturing system. Material supply, printer capability, inspection, repair, digital traceability, logistics, and recycling all influence sustainability. Industrial translation will depend on integrating these elements rather than optimizing the printer in isolation.

## 8. Technology Readiness, Research Gaps, and Integrated Design Guidelines

### 8.1. Cross-Cutting Research Priorities

Across both applications, there is a need for life-cycle assessments built around application-specific functional units. For pallets, useful units could be based on a defined payload moved over a service life or across a number of logistics cycles. For feed spacers, useful units could be treated water volume at a specified quality or membrane productivity over time. Such definitions allow material, print energy, transport, cleaning, replacement, and recovery to be compared on a common basis [5,10].

Techno-economic analysis should be conducted alongside environmental assessment. AM can eliminate tooling and enable customization, but machine time and material cost remain important. The break-even point depends on production volume, part size, complexity, customization value, service-life benefit, and the cost of failures or downtime. Sustainability claims are more credible when accompanied by a realistic pathway for manufacturing, qualification, maintenance, and end-of-life recovery.

Adoption barriers should also be recognized. Sustainable innovation can be slowed by supply-chain conservatism, uncertain standards, feedstock qualification, cost, process variability, and limited confidence in long-term performance [39]. These barriers are particularly relevant to pallets because logistics systems prioritize reliability and interchangeability, and to membrane components because water-treatment operators require chemically stable and predictable materials. Research should therefore include stakeholder and qualification requirements rather than treating printing capability as equivalent to industrial readiness.

The large number of interacting geometry, material, and process variables makes both application classes suitable for data-informed optimization. Artificial neural networks (ANNs), tree-based models, support-vector methods, Gaussian-process surrogates, and other machine-learning (ML) approaches can map nonlinear relationships among print parameters, dimensional error, mechanical response, energy, and defects; recent AM reviews also document their use for sensor-based anomaly detection and process-quality prediction [33,53]. Their main value in sustainable AM is not algorithmic complexity itself but the ability to reduce failed builds, narrow experimental search space, and support multi-objective optimization.

For pallet-scale structures, ML can link topology or lattice descriptors, infill, bead direction, material grade, recycled fraction, and process history to mass, stiffness, impact tolerance, creep, build time, and energy. For feed spacers, surrogate models can connect channel height, filament diameter, pitch, curvature, surface condition, and operating variables to pressure-drop gradient, shear, flux, fouling, and cleaning response. Physics-based constraints should be retained wherever possible so that an optimization does not recommend geometries that are unprintable, structurally unsafe, or hydraulically unrealistic [33,62,83].

ANN/ML models also introduce risks that are especially important in a review concerned with sustainability. Small datasets, leakage between training and test sets, machine-to-machine domain shift, changing recycled-feedstock properties, and black-box extrapolation can create apparently high predictive accuracy without reliable industrial transfer. Model reporting should therefore include dataset size, feature definition, train/validation strategy, uncertainty, external or confirmation testing, and the energy/material cost of generating the training data. ML should be used as a decision-support layer alongside mechanics, hydrodynamics, and lifecycle constraints rather than as a substitute for them [33].

Process monitoring is equally important. Vision, thermal, acoustic, force, or machine-state data can be used to detect incomplete fusion, under-cure, warpage, resin or feedstock instability, and dimensional drift before an entire build is rejected. Industry 4.0 integration provides the digital thread linking material lot, machine, software, parameter history, inspection, and acceptance data [33,35]. The integrated characterization, qualification, and translation requirements are consolidated in Table 5.

### 8.2. Technology-Readiness Interpretation

Technology readiness differs sharply between the two applications. FDM and large-format extrusion are mature manufacturing processes with a broad structural-material base, but the reviewed evidence does not yet establish a qualified whole AM pallet. The readiness gap is therefore application qualification, scale, build economics, creep/impact durability, and recycled-feedstock control rather than the basic ability to extrude thermoplastic. SLA and DLP are less credible for whole pallet-scale structures and are better positioned for small high-value inserts or design validation.

For membrane feed spacers, the pattern is almost reversed. Additive manufacturing has already produced direct laboratory-scale spacer evidence, including quantified changes in pressure drop, flux, and fouling (Table 4). The main readiness barriers are material qualification, long-term leaching and chemical stability, repeated cleaning, standardized hydrodynamic benchmarking, and module-scale translation. DLP/SLA are well matched to fine geometric exploration, while FDM remains useful where larger features or accessible prototyping are sufficient.

The combined evidence also supports hybrid technology pathways. A design may be developed in DLP for rapid high-resolution screening, transferred to FDM or another scalable polymer process for larger prototypes, and ultimately manufactured by a conventional high-throughput route if volume justifies tooling. Such a pathway does not diminish the value of AM. It recognizes AM as a design-discovery and flexible-production technology whose greatest contribution may occur at different stages of the product lifecycle.

The central conclusion is therefore not a ranking of SLA, DLP, and FDM. Each occupies a different region of the resolution–throughput–material–circularity design space, and its sustainability depends on whether that capability produces measurable service value. Process selection should follow the dominant functional requirement and the evidence quality available for the intended scale.

### 8.3. Integrated Design Rules

The combined evidence supports a compact set of design rules. Begin with the service function and its functional unit; select the AM process only when its geometric or material capability is necessary; optimize geometry and process parameters together; verify the as-printed geometry and material state; quantify energy, post-processing, service life, repair, and recovery rather than manufacturing mass alone; and avoid geometric complexity that does not produce a measurable structural or hydrodynamic benefit. Recycled or bio-derived content should be qualified through property retention and lifecycle metrics, while AI/ML optimization should remain constrained by physics and confirmed experimentally [5,10,33,34].

Research-scale feasibility must also be separated from industrial readiness. A feed spacer that performs well in a small cell still requires module-scale validation, long-duration fouling and cleaning studies, and water-contact qualification. A pallet-scale AM concept that carries a static load still requires repeated handling, creep, impact, environmental conditioning, sanitation where relevant, and compatibility with logistics systems. Life-cycle assessment and techno-economic analysis should accompany this qualification so that environmental gains are not achieved at unrealistic cost or through disproportionate process energy.

**Figure 8:**
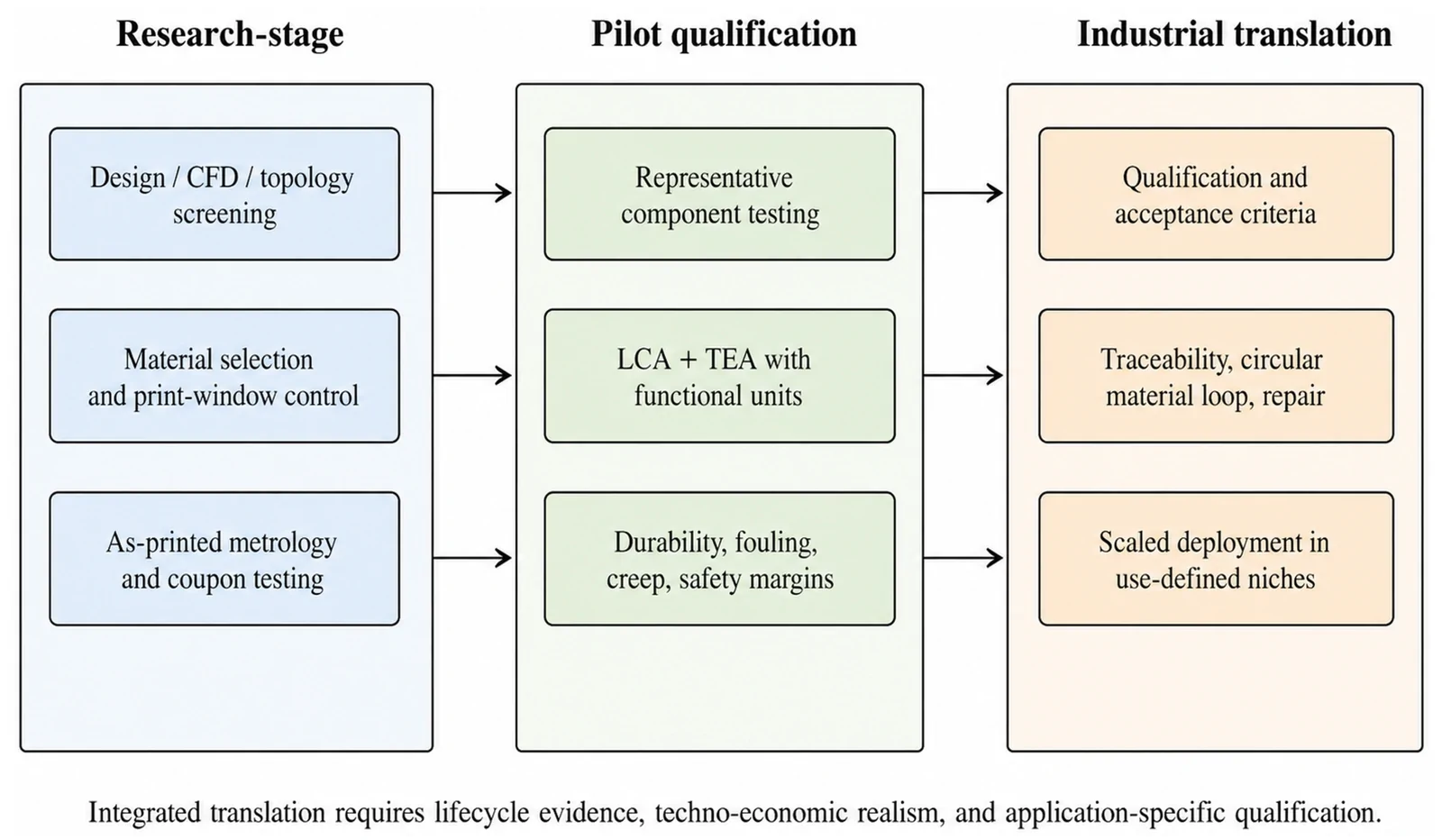
Integrated roadmap from research-stage design exploration to pilot qualification and industrial translation.

A robust translation pathway therefore proceeds from service definition to process/material selection, as-printed verification, representative component testing, lifecycle and techno-economic assessment, qualification, and finally deployment in use-defined niches. Figure 8 integrates these stages. Complexity should be introduced only when it produces measurable structural or hydrodynamic benefit, and recycled or renewable content should be evaluated alongside durability, quality retention, and end-of-life recovery. The quantitative tables in this review provide the evidence base needed to move this pathway from a qualitative process-matching argument toward measurable design decisions [1,2,3,4,5,35,39,51,52,53,60,64,88,132,133,134,135,137,138,139].

## 9. Conclusions

Polymer additive manufacturing can support sustainable production, but its environmental value is conditional rather than inherent. Across SLA, DLP, and FDM, the result depends on process energy, material production, dimensional fidelity, build yield, post-processing, service performance, durability, repair, supply-chain configuration, and end-of-life recovery. Quantitative evidence also shows why comparisons must preserve their functional units: desktop studies report markedly different energy intensities across machines and vat/extrusion processes, while upstream resin and recycling burdens can be substantial enough to change the lifecycle result [9,51,52,60,64].

For pallet-scale structures, the revised review deliberately moderates its claims. The cited evidence base does not establish a fully qualified additively manufactured transportation pallet. Instead, large-format FDM, structural polymer AM, cellular architectures, and recycling studies support a plausible translation pathway based on zoned load paths, local reinforcement, modular repair, controlled recycled feedstock, and hybrid manufacturing. The next evidence needed is full or representative pallet-scale validation under bending, fork impact, repeated handling, creep, environmental conditioning, build-rate economics, and closed-loop material control.

For membrane feed spacers, the evidence is more direct. Representative studies report pressure-drop reductions of 50–61% for perforated concepts, approximately threefold pressure-drop reduction with doubled specific water flux for a column spacer, a pressure-drop gradient of 0.091 ± 0.009 bar m^−1^ for a hole-pillar design under reported conditions, and a 16% permeate-flux increase with a substantially thinner fouling layer for a honeycomb geometry [128,132,133,134]. These results confirm that geometry can be an active operating variable, but long-term leachability, ageing, repeated cleaning, standardized baselines, and module-scale validation remain essential.

The strongest future direction is integrated, evidence-aware optimization. Geometry, material state, print parameters, as-printed metrology, service performance, lifecycle impact, cost, and uncertainty should be treated as one design problem. AI/ML can accelerate this search when datasets and validation are transparent, but it cannot replace mechanics, hydrodynamics, or lifecycle accounting. Sustainable polymer AM is most credible when the process is chosen because it creates a quantified service benefit that persists long enough to justify the resources used to manufacture, maintain, and recover the component.

## Figures and Tables

**Table 1:** Comparative assessment of SLA, DLP, and FDM, including dimensional/detail capability and the principal sustainability trade-offs.

Criterion	SLA	DLP	FDM/FFF
Primary mechanism	Laser-scanned vat photopolymerization	Whole-layer projected photopolymerization	Thermoplastic material extrusion
Typical detail-making ability	Very high; suited to fine channels and smooth surfaces	Very high; efficient for repeated fine features and arrays	Moderate; controlled by nozzle/bead size and stair-stepping
Main dimensional-accuracy controls	Layer thickness, laser spot, exposure, shrinkage, support and post-cure	Pixel/projection scale, exposure, cure depth, resin optics, shrinkage	Nozzle/bead width, layer height, thermal contraction, raster path, warpage
Throughput/scale tendency	High detail but scanning time rises with exposed path/area	Layer time is less sensitive to feature count; finite projection area	Highest structural scale potential through large-format or pellet extrusion
Typical sustainability strength	Precision can reduce machining/material excess; emerging bio-based resins	High feature density per layer and rapid small-part arrays	Thermoplastic repair/reprocessing, accessible equipment, structural scalability
Primary sustainability burden	Reactive resin, wash/post-cure, thermoset end-of-life	Resin chemistry, wash/post-cure, cured-resin recovery, projection energy	Anisotropy, thermal management, emissions, long large builds, feedstock variability
Pallet-scale role	Prototype or specialized high-detail subcomponent	Small high-value insert or sensor/identification feature	Most credible near-term route for structural-scale concepts, especially LFAM
Feed-spacer role	High-resolution research and complex channels	Very high suitability for repeated fine spacer geometries	Useful when coarser geometry is acceptable or larger prototypes are needed
Qualification priority	Long-term mechanical/chemical stability and leachability	Measured dimensions, leachability, cure, ageing and cleaning durability	As-printed geometry, interlayer integrity, emissions, durability and recycled-feedstock control

**Table 2:** Pallet-scale design requirements, AM-enabled response strategies, and evidence still required for validation.

Requirement	AM-Enabled Strategy	Key Risk Requiring Validation
Global stiffness	Ribbed decks, topology optimization, graded infill	Creep and anisotropic bending response
Local fork impact	Dense local reinforcement, sacrificial inserts, tough polymers	Interlayer delamination and notch sensitivity
Low mass	Cellular cores, TPMS/lattice regions, hollow beams	Buckling and local crushing
Durability	Engineering thermoplastics, protected edges, modular repair	Environmental ageing and repeated-load damage
Cleanability	Smooth external skins, drain paths, reduced inaccessible cavities	Porosity and contamination retention
Circularity	Mono-material architecture, take-back, regrinding, replaceable modules	Property drift in recycled feedstock
Digital logistics	Integrated sensor/RFID housings and identifiers	Electronic separation at end of life

**Table 3:** Feed-spacer design variables, intended hydrodynamic benefits, and principal penalties or risks.

Design Variable	Potential Benefit	Potential Penalty/Risk
Filament diameter and blockage	Greater flow perturbation and mechanical spacing	Higher pressure drops and reduced open area
Crossing angle/orientation	Controls vortices, flow redistribution, and shear	May create stagnation zones or high local losses
Surface texture	Boundary-layer disruption and mixing	Foulant attachment and difficult cleaning
Curvature/sinusoidal paths	Secondary flow and smoother directional changes	Higher geometric complexity and print sensitivity
Graded architecture	Local mixing only where needed	More difficult optimization and validation
TPMS-like geometry	Continuous smooth pathways and high design freedom	Complex metrology and scale-up
Material chemistry	Chemical resistance or tailored surface properties	Leaching, swelling, brittleness, or recycling difficulty

**Table 4:** Quantitative evidence from representative feed-spacer studies and relevant comparators.

Study	Printing Method/Material	Geometry/Channel height/Filament Dimensions	Pressure-Drop/Hydraulic Result	Flux/Fouling/Cleaning Result
Siddiqui et al. [115]	PolyJet 3D printing; printed reference-matched and modified feed spacers	Practical/reference spacer thickness range 26–34 mil (0.66–0.86 mm); printed strand thicknesses across adopted geometries were reported in the same approximate range; mesh angle and spacing were varied in the modified design	The printed spacer reproducing the practical geometry showed similar hydraulic behaviour to the reference; the modified printed geometry reduced pressure-drop development	The modified geometry reduced biofouling impact and established a combined modelling–printing–MFS workflow for spacer development
Lin et al. [125]	Commercial non-uniform filament spacers; experimental + numerical comparator (not an AM study)	Non-uniform filament geometry; study-specific channel and filament dimensions	Geometry altered feed-channel hydraulic resistance and pressure-drop development; retained here as a comparator for AM design targets	Anti-fouling response depended on the non-uniform filament architecture; useful for separating geometry effects from printing effects
Kerdi et al. [128]	High-resolution 3D printing (MiiCraft 125); photopolymer perforated spacers	Symmetric 0-, 1-, 2-, and 3-hole designs; strand angle α = 45° and incident-flow angle β = 90°; holes introduced through cylindrical filaments	The 3-hole spacer produced about 50–61% lower pressure drop than the 0-hole printed baseline over the tested velocities	The 1-hole spacer increased steady permeate production by about 75% at constant pressure and 23% at constant feed flow; it also produced the cleanest membrane surface
Ali et al. [133]	High-resolution vat photopolymerization (MiiCraft 125); acrylate-resin column-type spacer	Flow-channel height H = 1.2 mm; reduced filament diameter ≈ 0.5 mm, column-node diameter ≈ 1.5 mm, node spacing ≈ 4 mm; α = 45°, β = 90°; clearance increased to ≈0.35 mm	Pressure drop was approximately three times lower than for the standard symmetric spacer	Specific water flux approximately doubled, biomass accumulation was markedly lower, and specific energy consumption was about twofold lower
Qamar et al. [132]	High-resolution 3D printing (MiiCraft 125); commercial-like, pillar, and hole-pillar spacers	Spacer/channel height H = 1.2 mm; coupons 60 × 15 mm; pillar/hole-pillar clearance ≈ 0.35 mm; feed velocity 0.185 m s^−1^ in the reported comparison	Pressure-drop gradients: commercial 0.118 ± 0.009, pillar 0.141 ± 0.007, and hole-pillar 0.091 ± 0.009 bar m^−1^	At 0.5 bar after 67 h, steady flux was 11.8, 14.6, and 20.7 L m^−2^ h^−1^ for commercial, pillar, and hole-pillar spacers, respectively; the hole-pillar design also produced the thinnest biofilm among the three
Park et al. [134]	3D-printed honeycomb feed-channel spacer; nanofiltration comparison with standard diamond spacer	Honeycomb architecture; geometry was study-specific and is not converted here into a common filament-diameter/pitch metric	Hydrodynamics were evaluated together with fouling using experiments and CFD rather than by pressure-drop ranking alone	Fouling layer thickness was 119.0 μm versus 175.5 μm for the standard spacer (≈33% lower); permeate flux was 16.0% higher and hydraulic cleaning recovery was improved

**Table 5:** Integrated characterization, qualification, and translation requirements for robust pallet-scale and feed-spacer AM studies.

Category	Pallet Studies	Feed-Spacer Studies
Material state and circularity	Polymer grade, recycled fraction, moisture/conditioning, reinforcement, multi-cycle reprocessing limits, contamination control, and material traceability	Resin/thermoplastic chemistry, cure state, water conditioning, recycled fraction where applicable, material-lot traceability, and compatibility with the intended water-contact environment
Process parameters and traceability	Layer height, nozzle/bead size, temperatures, raster/infill, orientation, machine state, material lot, and digital build record	Layer/exposure settings or nozzle geometry, pixel/nozzle scale, orientation, post-cure, resin/material lot, and digital build record
As-printed geometry and metrology	Warp, section dimensions, internal architecture, roughness, porosity/defect mapping, and geometry uncertainty	Filament/channel dimensions, open area, roughness, blocked features, defect mapping, and geometry uncertainty
Application performance	Static, impact, cyclic, creep, rack/fork-entry and repeated-handling response under representative service conditions	Pressure-drop gradient, mass transfer or flux, fouling evolution, cleaning recovery, and operating-condition sensitivity
Long-term durability	Repeated handling, temperature/humidity conditioning, wear, creep retention, ageing, and repair/reuse performance	Long immersion, repeated chemical cleaning, swelling, dimensional stability, leaching, and fouling/cleaning-cycle durability
Safety and material qualification	Material identity, relevant emissions/occupational exposure, chemical compatibility, and sanitation requirements where applicable	Degree of cure/conversion, extractables/leachables, swelling, long-term water-contact stability, and chemical-cleaning compatibility
Statistics and reproducibility	Replicates, variability, build-to-build repeatability, process-window robustness, and uncertainty reporting	Replicates, hydraulic uncertainty, geometry–performance variation, build-to-build repeatability, and uncertainty reporting
Model, AI/ML, and digital validation	Process-aware material properties, component correlation, transparent datasets, uncertainty, confirmation tests, and cross-machine validation	Measured geometry linked to pressure-drop/flow data, transparent datasets, uncertainty, confirmation tests, and cross-machine validation
Application-specific qualification standards	Representative structural protocols, service-relevant baselines, acceptance limits, and staged coupon-to-component-to-pallet qualification	Representative hydraulic/module protocols, cleaning and ageing baselines, acceptance limits, and staged cell-to-module qualification
Lifecycle and techno-economics	Functional-unit LCA/TEA including machine utilization, post-processing, service life, repair, logistics, recycling, and end of life	Functional-unit LCA/TEA including printing/post-cure, pumping energy, cleaning, membrane life, replacement, recovery, and end of life

**Table 6:** Quantitative energy and environmental benchmarks relevant to polymer AM sustainability.

Source	Process/Stage	Functional Unit or Test Context	Reported Value	Critical Interpretation
Suárez and Domínguez [8]	FDM sustainability review	Review-level evidence across FDM studies	No single transferable SEC; energy depends strongly on machine, build time, utilization, material, and process settings	Included to define the heterogeneity of FDM energy studies; review values should not be treated as one process constant
Faludi et al. [137]	FDM vs. PolyJet vs. CNC life-cycle assessment	Cradle-to-grave manufacture of two benchmark polymer parts; high- and low-utilization scenarios	FDM had the lowest ecological impact per part among the three machines in both utilization scenarios; electricity dominated the impacts of both 3D printers	Utilization and idle energy can reverse or magnify comparisons; this is an LCA result, not an absolute SEC for all FDM systems
Song and Telenko [139]	Commercial FDM use phase	Energy and material loss normalized by polymer throughput in a heavily utilized open shop	Preheat 0.835 MJ kg^−1^ (0.232 kWh kg^−1^); printing 21.5 MJ kg^−1^ (5.97 kWh kg^−1^); standby 9.5 MJ kg^−1^ (2.64 kWh kg^−1^); actual energy LCI ≈ 50% above ideal; ≈34% of plastic used was wasted	Failed builds, calibration, standby, orientation, and support strategy are sustainability variables, not merely quality-control issues
Hopkins et al. [51]	FFF	Desktop printers; useful printed volume	24.8–85.7 kJ cm^−3^	Machine, bed temperature, layer height, and operating state strongly affect the result
Hopkins et al. [51]	SLA	Desktop vat photopolymerization; useful printed volume	10.8–21.5 kJ cm^−3^	Printing energy alone does not include all washing, solvent, and post-cure burdens
Hopkins et al. [51]	Mask-SLA/area-exposure vat photopolymerization	Desktop area-exposure system; useful printed volume	18.4–19.3 kJ cm^−3^	Useful area-exposure benchmark relevant to DLP-type comparisons, but not interchangeable with every DLP platform
Lunetto et al. [52]	FDM	149.5 g validation build; 301 min	9.47 MJ = 2.63 kWh per build; modelled 66.2 MJ kg^−1^ = 18.4 kWh kg^−1^	Specific energy decreased as useful deposition rate increased under the studied conditions
Yi et al. [140]	Eco-design energy assessment	AM energy-performance framework applied at design/process level	Framework reports energy-performance indicators for the selected use case rather than a universal process SEC	Useful for linking parameter selection and eco-design; absolute values remain machine-, geometry-, and boundary-specific
Woern and Pearce [141]	Polymer pelletizing/chopping for recycled feedstock preparation	Open-source 3-D printable pelletizer chopper	0.24 kWh kg^−1^ during chopping; throughput 1.0 kg h^−1^ with two motors	Quantifies feedstock-preparation energy rather than printer build energy and reinforces the importance of clearly defining the system boundary
Ahire et al. [60]	Lignin-blended resin production	10 wt% lignin blend; resin-production boundary	6.64 kg CO_2_-eq kg^−1^ resin; production cost $38.43 kg^−1^	Quantifies upstream material burden; does not represent the complete printed-part lifecycle
Xie et al. [64]	Conventional DLP comparator in multi-material study	Relative comparison with volumetric photopolymerization for the same structure	Volumetric route used about 1/2–1/6 of DLP energy and 1/9–1/22 of DLP fabrication time	Relative benchmark demonstrates sensitivity to exposure strategy; it is not a universal absolute DLP SEC

**Table 7:** Quantitative recycled-polymer trade-offs relevant to FDM circularity.

Source/Material	Recycling Condition	Mechanical or Surface Response	Environmental/Processing Response	Design Implication
Yang et al. [7]	Virgin and recycled FDM filament; multi-objective parameter optimization	Mechanical retention was not the primary reported endpoint; orientation, layer height, and heated-bed temperature were the most influential recycled-filament variables	Optimized settings improved the combined objectives by 20.24%, reduced particulate-matter impact by 54.02%, and reduced lifecycle emissions by 21.7%	Environmental advantage from recycled filament depends on the printing window; sustainability cannot be separated from parameter optimization
Olawumi et al. [9]	Repeated recycling of polymer composites	Up to 90% tensile-strength retention after the first cycle; about 80% after three cycles	Recycling-process energy decreased 30%; carbon footprint decreased 25% in the reported study	Repeated recycling can be viable, but property retention should be tracked by cycle and polymer system
Pinho et al. [87]	Post-consumer PLA and ABS	Recycled PLA: ≈33% loss in tensile stress and flexural strength; recycled ABS: no meaningful mechanical loss; roughness decreased 55–65%	Post-consumer polymers were reintroduced into functional FDM parts; lifecycle benefit depends on collection and reprocessing boundary	Polymer identity strongly changes the performance penalty; recycled content should not be generalized across materials
Hasan et al. [88]	0–100% post-consumer rPLA blended with virgin PLA	100% rPLA: tensile −48.4%, flexural −49%, roughness +53.73%; blends up to 50% rPLA retained >90% flexural performance	Extends post-consumer PLA use; study emphasizes resource efficiency rather than a complete LCA	Controlled blending can provide a better performance–circularity compromise than 100% rPLA
Woern and Pearce [141]	Waste-polymer pellet preparation for FGF/FPF and subsequent recyclebot use	High-tolerance thermopolymer pellets; throughput of 0.5 kg h^−1^ with one motor and 1.0 kg h^−1^ with two motors	0.24 kWh kg^−1^ during chopping; open-source pelletizer system reported at approximately US$185	Distributed feedstock preparation can reduce recycling barriers, but feedstock quality and subsequent printed-part qualification remain separate requirements

## Data Availability

No new data were created or analyzed in this study. Data sharing is not applicable to this article.
